# Illuminating Mitochondrial RNA G‐Quadruplexes as Structural Brakes on RNA Granule Assembly and OXPHOS

**DOI:** 10.1002/advs.202523462

**Published:** 2026-02-12

**Authors:** Gui‐Xue Tang, Jia‐Tong Yan, Mao‐Lin Li, Cui Zhou, Jian Wang, Shuo‐Bin Chen, Zhi‐Shu Huang, Jia‐Heng Tan

**Affiliations:** ^1^ State Key Laboratory of Oncology in South China Sun Yat‐sen University Cancer Center Guangzhou China; ^2^ School of Pharmaceutical Sciences Sun Yat‐sen University Guangzhou China

**Keywords:** GRSF1, mitochondrial RNA G‐quadruplex, mitochondrial RNA granule, molecular probe, Wnt/β‐catenin pathway

## Abstract

G‐quadruplexes (G4s) have been extensively investigated in cells, with established methods available for studying nuclear DNA G4s, cytoplasmic RNA G4s, and even mitochondrial DNA G4s. However, mitochondrial RNA (mtRNA) G4s have remained largely unexplored in cells due to the lack of suitable tools, leaving their biological functions poorly understood. Here, through rational molecular design, we developed **MitoQUMA**, a fluorescent probe that allows the visualization of mtRNA G4 dynamics in live cells. Using this probe, we observed that, unlike cytoplasmic RNA G4s, which generally promote phase separation to form RNA granules, excessive formation of mtRNA G4s correlates with reduced assembly of mitochondrial RNA granules (MRGs). A **MitoQUMA**‐based chemical genetic screen revealed that the Wnt/β‐catenin pathway regulates this mitochondrial event by modulating GRSF1 expression, thereby affecting mtRNA G4 abundance and processing. When RNA processing is compromised, mtRNA maturation is impaired, and MRG becomes unstable and undergoes disassembly, ultimately disrupting mitochondrial gene expression and energy metabolism. Collectively, our study introduces a tool for real‐time monitoring of mtRNA G4s in cells and identifies the Wnt/β‐catenin–GRSF1–mtRNA G4 axis as a previously unrecognized pathway coordinating MRG assembly and energy metabolism, providing new insights into phase separation within mitochondria.

## Graphical abstract

A new fluorescent probe, **MitoQUMA**, brings mitochondrial RNA G‐quadruplexes (mtRNA G4s) into view in live cells. This tool reveals that excessive mtRNA G4 formation halts mitochondrial RNA granule (MRG) assembly, opposite to the phase‐separation role of cytoplasmic RNA G4s. A chemical genetic screen further identifies a Wnt/β‐catenin–GRSF1–mtRNA G4 axis linking mtRNA processing and MRG control to OXPHOS.

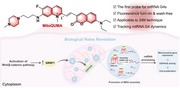

## 1 Introduction

Mitochondria are central hubs of cellular bioenergetics and metabolism, not only generating ATP through oxidative phosphorylation (OXPHOS) but also orchestrating essential processes such as apoptosis regulation and immune responses [1]. Proper mitochondrial function depends on the precise expression of the mitochondrial genome (mtDNA). Human mtDNA is a double‐stranded, closed circular molecule of 16 569 bp that encodes 37 genes, including two ribosomal RNAs, 22 transfer RNAs, and 13 messenger RNAs. In addition, mtDNA encodes various noncoding RNAs (e.g., 7S RNA and lncND5) [2, 3]. Collectively, these mitochondrial RNAs (mtRNAs) contribute to the expression of the core OXPHOS components localized within the inner mitochondrial membrane. Dysregulation of mitochondrial gene expression is closely linked to the onset and progression of numerous diseases. Therefore, elucidating the mechanisms that regulate mtRNA expression has become a central focus in chemistry, life sciences, and medical research.

Among the structural elements that influence RNA metabolism and gene expression, G‐quadruplexes (G4s) have attracted considerable attention [4, 5, 6]. These noncanonical nucleic acid structures are formed from guanine‐rich (G‐rich) sequences that fold into stable four‐stranded conformations through Hoogsteen hydrogen bonding. Bioinformatic analyses indicate that the mitochondrial genome contains abundant potential G4‐forming sequences (PQSs) distributed across both coding and noncoding regions [7, 8]. During transcription, G‐rich mtRNAs derived from the complementary C‐rich strand exhibit a strong tendency to form G4s. Previous studies suggest that PQSs in mtRNAs are particularly enriched within noncoding RNAs, where the RNA‐binding protein GRSF1 resolves G4s to promote RNA turnover and sustain mitochondrial translation and OXPHOS [9].

**FIGURE 1:**
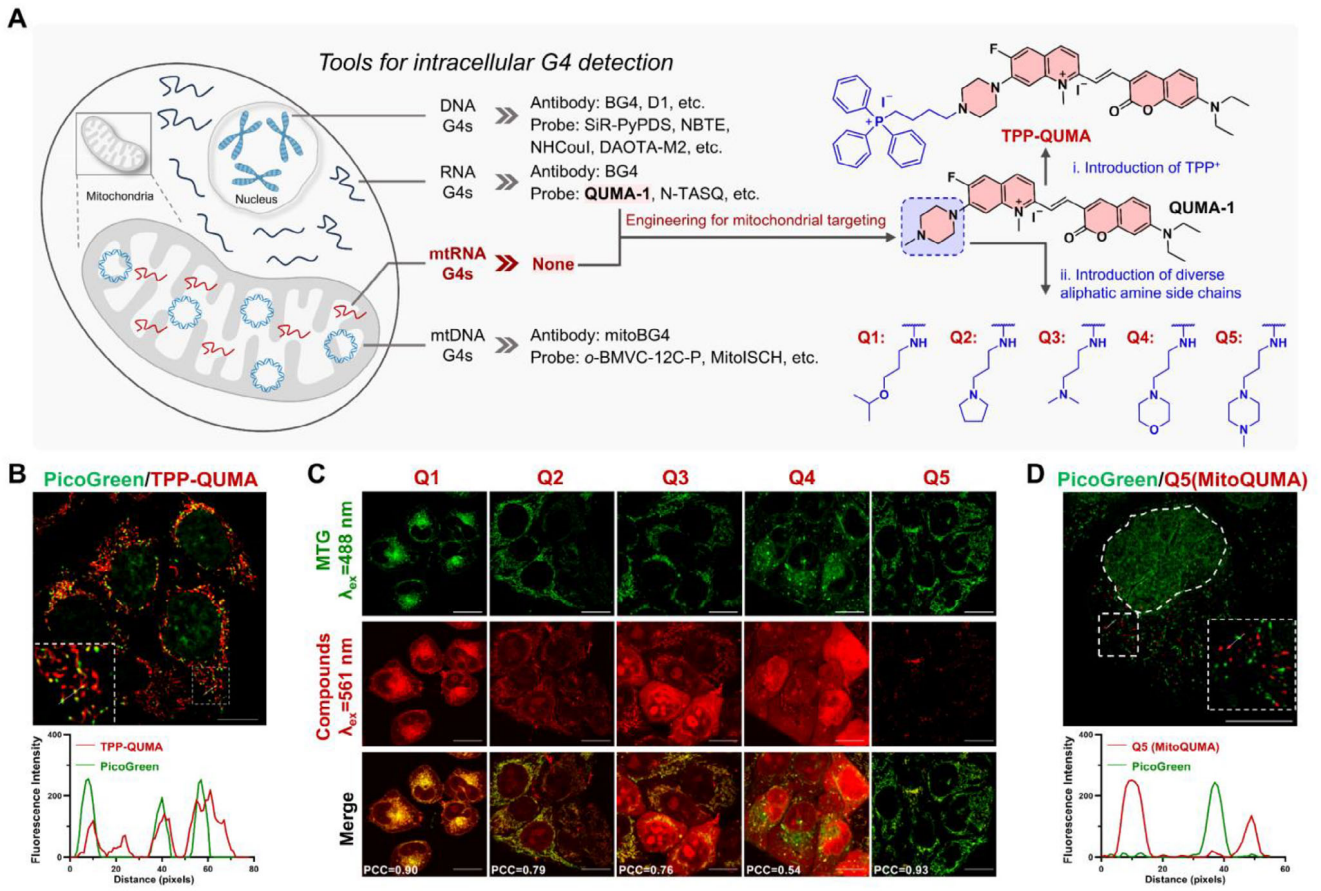
Rational design of fluorescent probes for live‐cell imaging of mtRNA G4s. (A) Engineering of QUMA‐1 for mtRNA G4 detection. (B) Confocal image of live HeLa cells co‐stained with 0.1 µm PicoGreen and 2 µm **TPP‐QUMA**. Fluorescence intensity profiles across the white line in the white box are shown. (C) Confocal image of live HeLa cells stained with 2 µm **Q1‐Q5** (λ_ex_ = 561 nm), and their mitochondria were labeled with 50 nm MitoTracker Green (MTG, λ_ex_ = 488 nm). Pearson's correlation coefficient (PCC) was used to quantify the degree of colocalization between **Q1‐Q5** and MTG in the cytoplasm. (D) Super‐resolution image of live HeLa cells co‐stained with 0.1 µm PicoGreen and 2 µm **Q5** (**MitoQUMA**). Fluorescence intensity profiles across the white line in the white box are shown. Scale bars for the cell image: 10 µm.

Despite these findings, most insights into mtRNA G4s have come from in vitro studies, and direct evidence in cells is still lacking. This limitation stems from the absence of effective tools to probe mtRNA G4s in their native cellular environment, leaving their physiological roles poorly understood. In contrast to nuclear DNA G4s [10, 11, 12, 13], cytoplasmic RNA G4s [14, 15, 16], and mitochondrial DNA G4s [17, 18, 19], for which molecular probes already exist, mtRNA G4s remain uniquely inaccessible (Figure 1A). Developing probes that can visualize and characterize mtRNA G4s in cells is therefore both a pressing need and a major challenge, with significant implications for understanding mitochondrial gene regulation and cellular metabolism.

In our previous work, we developed QUMA‐1, a small‐molecule fluorescent probe capable of specifically detecting cytoplasmic RNA G4s [14]. Using this probe, we were able to visualize RNA G4 dynamics in live cells, capturing their folding and unfolding processes as well as interactions with G4‐binding proteins. Subsequently, several research groups employed QUMA‐1 to investigate the biological functions of RNA G4s in cells, yielding important advances. These studies validated the robust performance of QUMA‐1, which has since been incorporated into Sigma‐Aldrich's BioTracker product line, making it one of the widely used commercial probes for RNA G4 research. However, QUMA‐1 is restricted to cytoplasm and cannot access mitochondria. To address the unmet need for mtRNA G4 probes, we envisioned using QUMA‐1 as a lead compound and rationally modifying its structure to enable mitochondrial localization. We selected QUMA‐1 based on our prior experience, in which rational structural modification of a DNA G4 fluorescent probe successfully enabled mitochondrial targeting while preserving selective recognition of mtDNA G4s [17]. This experience suggested that QUMA‐1 represents a suitable starting point for the development of fluorescent probes targeting mtRNA G4s. The development of such a probe would provide a powerful molecular tool for investigating the biological functions of mtRNA G4s, offer deeper insights into their roles in mitochondrial gene expression, and potentially guide the discovery of novel therapeutic targets.

## 2 Results and Discussion

### 2.1 Rational Design of Fluorescent Probes for Live‐Cell Imaging of mtRNA G4s

Mitochondria are characterized by a phospholipid bilayer and negative membrane potential (approximately −150 to −180 mV), so small molecules targeting mitochondria typically require high lipophilicity together with a delocalized positive charge [20]. Although the parent scaffold of QUMA‐1 carries a positive charge, it fails to exploit the mitochondrial membrane potential for effective accumulation, likely due to insufficient lipophilicity, low charge density, or a combination of both. Current strategies for mitochondrial targeting include the use of mitochondrial‐targeting peptides or lipophilic cations. Considering that bulky peptides may interfere with probe‐mtRNA G4s interactions, we chose to introduce a lipophilic cation. The triphenylphosphonium (TPP^+^) moiety is the most widely used mitochondriotropic carrier [21]. Based on our previous finding that the coumarin core of QUMA‐1 is critical for specific RNA G4 recognition, we incorporated TPP^+^ at the C2 side chain of QUMA‐1, generating **TPP‐QUMA** (Figure 1A). Unexpectedly, unlike our previous success in developing TPP^+^‐conjugated probes targeting mtDNA G4s [17], live‐cell imaging revealed that although **TPP‐QUMA** localized to mitochondria, part of its punctate signal colocalized significantly with PicoGreen, an mtDNA indicator (Figure 1B). This suggests that the high positive charge density introduced by the TPP^+^ moiety enhanced binding to mtDNA rather than selectively targeting mtRNA.

We therefore revised our modification strategy to adjust both the lipophilicity and the effective positive charge of QUMA‐1 using alternative substituents. It has been reported that elongated alkyl side chains can enhance mitochondrial uptake [22]. Accordingly, we introduced a series of aliphatic amine side chains with varying lipophilicity and protonation potential at the C2 position of QUMA‐1, generating five derivatives, **Q1‐Q5** (Figure 1A). The detailed synthetic procedures for **TPP‐QUMA** and **Q1**‐**Q5** are provided in the (Scheme S1).

Next, we evaluated whether these derivatives could enter mitochondria and target mtRNA G4s in live cells using confocal microscopy. As shown in Figure 1C, while all five compounds entered mitochondria, **Q5** showed distinct punctate signals, with a PCC value of 0.93. The other derivatives produced diffuse staining or induced alterations in cellular morphology. Structure‐activity analysis revealed that **Q1**, bearing a three‐methylene alkyl chain, enhanced lipophilicity but lacked a terminal protonatable nitrogen atom that may be important for G4 recognition, and was associated with pronounced effects on cellular state. **Q2**‐**Q4**, featuring protonatable amino groups at the chain termini, localized mainly to both the nucleus and mitochondria, indicating that their lipophilicity and charge remained suboptimal for precise mitochondrial targeting. In contrast, **Q5** incorporated a long alkyl chain terminating in an N‐methylpiperazine moiety, similar to QUMA‐1, which not only provided a protonatable site to enhance positive charge but also increased lipophilicity, thereby conferring superior mitochondrial specificity. The mitochondrial matrix localization of **Q5** was further confirmed by super‐resolution structured illumination microscopy (Figure S1A). Importantly, both confocal and super‐resolution imaging showed negligible colocalization between PicoGreen and **Q5** (Figure 1D; Figure S1B), indicating minimal mtDNA interaction. Collectively, these results support the selection of **Q5** as a candidate probe for mtRNA G4s, which we subsequently designated as **MitoQUMA**.

### 2.2 Evaluation of MitoQUMA as a Fluorescent Probe for mtRNA G4s

**FIGURE 2:**
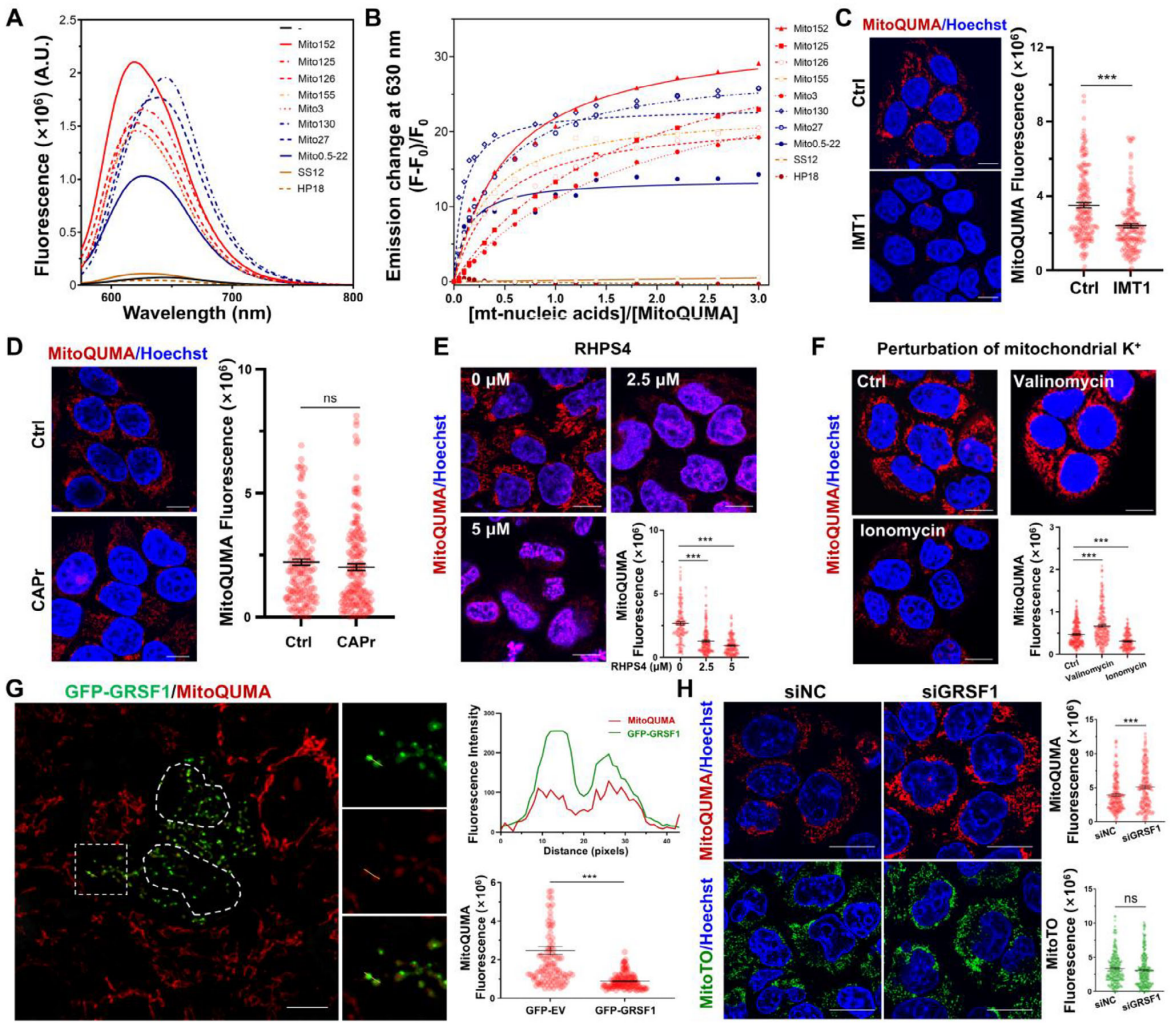
Evaluation of MitoQUMA as a fluorescent probe for mtRNA G4s. (A) Fluorescence emission spectra of 1 µm **MitoQUMA** with or without 3 µm of different mtRNAs (mtRNA G4s: Mito152, Mito125, Mito126, Mito155, Mito3; double‐stranded mtRNA: HP18; single‐stranded mtRNA: SS12) and mtDNA G4s (Mito130, Mito27, Mito0.5‐22). (B) Fluorescence emission change of 1 µm **MitoQUMA** at 630 nm vs. [mt‐nucleic acids]/[**MitoQUMA**]at λ_ex_ = 555 nm. (C–F) Live HeLa cells were treated with 5 µm IMT1 (C), 200 µg/mL chloramphenicol (CAPr) (D), varying concentrations of RHPS4 (E), valinomycin (10 µm together with 200 mm KCl) or ionomycin (20 µm together with 20 mm KCl) (F), and stained with 2 µm **MitoQUMA**. (G) Confocal image of live HeLa cells with overexpression of GFP‐tagged GRSF1 were stained with 2 µm **MitoQUMA**. Fluorescence intensity profiles across the yellow line in the white box were shown. (H) Live HeLa cells were transfected with siRNA to knock down GRSF1 expression, followed by staining with 2 µm **MitoQUMA**. For each sample of cell image, approximately 100 cells were measured. Biological replicates (*n* = 3) were taken. The data are presented as mean ± SEM, and statistical significance is determined by the two‐sided Student's unpaired *t‐*test (C, D, G, and H) and one‐way ANOVA followed by Turkey's multiple‐comparison test (E and F) as (ns) not significant, (^*^) *p* <0.05, (^**^) *p* < 0.01, and (^***^) *p* < 0.001. Scale bars for cell image: 10 µm.

Because **MitoQUMA** did not show obvious colocalization with PicoGreen‐labeled mtDNA during the initial cellular screening, we first performed fluorescence spectroscopy assays to evaluate its interaction with different mtRNA structures (Table S1 and Figure S2). As shown in Figure 2A, **MitoQUMA** alone in buffer displayed negligible fluorescence emission. Upon addition of mtRNA G4s, a pronounced fluorescence enhancement at 630 nm was observed, whereas the addition of single‐stranded or double‐stranded mtRNA induced comparatively weaker signal changes (Figure 2B). These results suggest that, among mtRNA species, **MitoQUMA** exhibits a higher response toward G4 structures in vitro. In addition to mtRNAs, we also examined its interaction with different mtDNA G4s under cell‐free conditions. We found that **MitoQUMA** responded slightly more strongly to certain mtRNA G4s than to mtDNA G4s. However, the overall differences between these responses remained modest. This behavior is consistent with our previous observations for the RNA G4 fluorescent probe QUMA‐1, which likewise displayed comparable interactions with nuclear DNA G4s and RNA G4s in cell‐free fluorescence spectroscopy assays. Based on these results and our prior experience, we propose that differences in the microenvironments occupied by mtRNA and mtDNA within mitochondria may contribute to the observed cellular behavior of **MitoQUMA**.

Next, we sought to further evaluate the performance of **MitoQUMA** for visualizing mtRNA G4s in live cells. Before cellular studies, we characterized the probe's basic properties relevant to its use in biological imaging. **MitoQUMA** exhibited robust photostability in both buffer solutions and intracellular environments, as well as low cytotoxicity following 24 h treatment (Figure S3). Optimization of imaging conditions in HeLa cells revealed that a 3‐h incubation with 2 µm **MitoQUMA** provided optimal fluorescence signal and imaging clarity (Figure S4) without appreciably perturbing G4 folding or unfolding dynamics or altering their topology, as assessed by CD melting and titration analyses (Figure S5).

Following these validations, we performed enzymatic digestion assays in fixed cells. RNase A treatment completely abolished **MitoQUMA** signals, whereas DNase I treatment had no significant effect, indicating that the probe primarily binds RNA in cells (Figure S6A). To verify the mitochondrial targets of **MitoQUMA**, we compared staining in wild‐type cells vs. ρ^0^ cells lacking mtDNA using **MitoQUMA** and MitoTO, a total mitochondrial nucleic acid dye previously developed by our group [17]. Both probes exhibited markedly reduced fluorescence in ρ^0^ cells compared to controls, with only a weak residual signal from **MitoQUMA** observed in rRNA‐enriched nucleolar regions (Figure S6B), consistent with mitochondrial nucleic acids being the primary targets of **MitoQUMA**. Furthermore, inhibition of mitochondrial RNA synthesis using the transcription inhibitor IMT1 significantly decreased **MitoQUMA** fluorescence without affecting the mtDNA indicator PicoGreen (Figure 2C; Figure S6C). In contrast, inhibition of mitochondrial translation using chloramphenicol did not alter **MitoQUMA** signals (Figure 2D). These results indicate that **MitoQUMA** preferentially associates with mtRNAs in cells.

To further assess the selectivity of **MitoQUMA** for mtRNA G4s, we performed competition experiments using the reported mitochondrial G4 ligand RHPS4 [23]. RHPS4 treatment reduced **MitoQUMA** punctate signals and overall fluorescence intensity (Figure 2E), suggesting competition for mitochondrial G4 binding sites. Because G4 formation is highly sensitive to local ionic conditions, especially K^+^ concentration, we also modulated mitochondrial K^+^ levels using the ionophores valinomycin (induces K^+^ influx) and ionomycin (induces K^+^ efflux) [24, 25, 26]. As shown in Figure 2F, increasing mitochondrial K^+^ via valinomycin enhanced **MitoQUMA** fluorescence, whereas ionomycin‐induced K^+^ efflux suppressed fluorescence. In a control experiment, mitochondrial K^+^ levels minimally affected MitoTO signals (Figure S6D), and similarly, examination of the mtRNA fluorescent probe Pyronin Y as an additional control showed that modulation of mitochondrial K^+^ levels did not produce pronounced changes in its fluorescence (Figure S6E).

Finally, we investigated the interplay between **MitoQUMA** signals and the quasi‐RNA recognition motif (qRRM) protein GRSF1, the only reported protein known to unwind mtRNA G4s [9]. Overexpression of GFP‐tagged GRSF1 markedly reduced **MitoQUMA** fluorescence and resulted in significant colocalization of red **MitoQUMA** signals with green GRSF1 foci (Figure 2G). Conversely, siRNA‐mediated knockdown of GRSF1 enhanced **MitoQUMA** fluorescence (Figure 2H; Figure S6F). In these experiments, the signals of the control probes MitoTO (Figure 2H) and Pyronin Y (Figure S6G,H) remained largely unchanged (MitoTO was not included as a control in the GFP‐GRSF1 overexpression experiments due to spectral overlap with GFP emission). Collectively, these findings demonstrate that **MitoQUMA** enables selective visualization of mtRNA G4s in live cells.

### 2.3 Mitochondrial RNA G4s Regulates the Assembly of Phase‐Separated Mitochondrial RNA Granules

**FIGURE 3:**
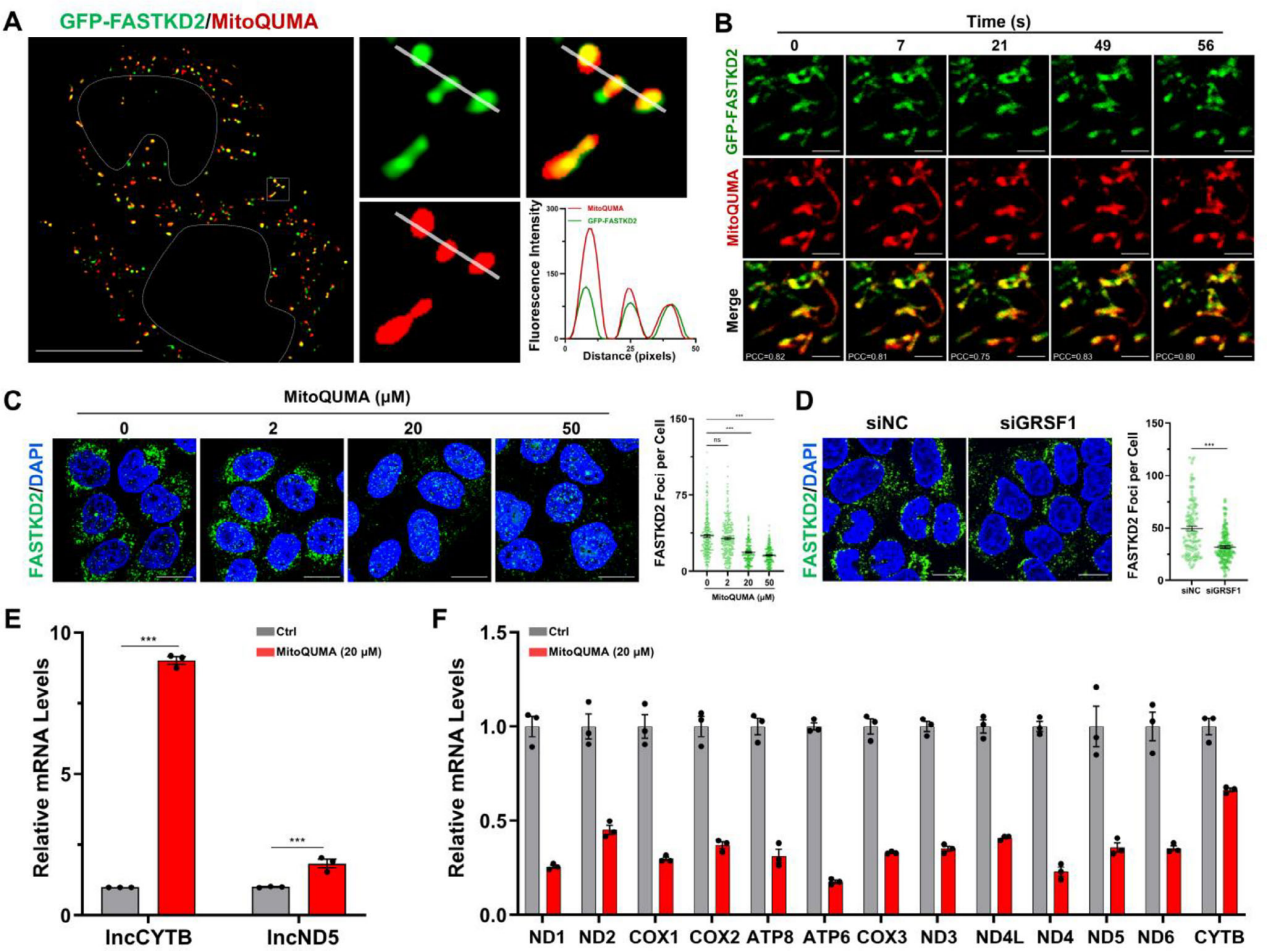
Mitochondrial RNA G4s regulates the assembly of phase‐separated MRGs. (A) Super‐resolution image of live HeLa cells with overexpression of GFP‐tagged FASTKD2 were stained with 2 µm **MitoQUMA**. Fluorescence intensity profiles across the white line in the white box were shown. (B) Dynamics of mtRNA G4s and MRGs (labeled with GFP‐FASTKD2), as measured by the time‐dependent movement of **MitoQUMA** (2 µm) foci in live HeLa cells imaged by confocal. Pearson's correlation coefficient (PCC) was used to quantify the degree of colocalization between **MitoQUMA** and GFP‐FASTKD2. (C) Effects of high‐dose **MitoQUMA** on MRG formation in HeLa cells. MRGs were indicated by immunofluorescence staining of FASTKD2. (D) Validation of decreased MRG formation in HeLa cells with siRNA‐mediated silencing of GRSF1. MRGs were indicated by immunofluorescence staining of FASTKD2. (E,F) L‐strand transcripts (lncCYTB and lncND5) and mitochondrial gene expression levels in HeLa cells treated with 20 µm **MitoQUMA**. For each sample of cell image, approximately 100 cells were measured. Biological replicates (*n* = 3) were taken. The data are presented as mean ± SEM, and statistical significance is determined by the one‐way ANOVA followed by Turkey's multiple‐comparison test (C), two‐sided Student's unpaired *t*‐test (D), and two‐way ANOVA with Sidak's multiple comparisons test (E) as (ns) not significant, (^*^) *p* < 0.05, (^**^) *p* < 0.01, and (^***^) *p* < 0.001. Scale bars for cell image of Figure 3A,C, and D: 10 µm. Scale bars for cell image of Figure 3B: 2 µm.

GRSF1 is a mitochondrial matrix‐localized RNA‐binding protein. While recent studies have implicated GRSF1 in the regulation of mtRNA G4 folding, its well‐established role is as a core component of mitochondrial RNA granules (MRGs) [27]. MRGs are dynamic, liquid‐liquid phase‐separated structures composed of newly transcribed mtRNAs and multiple RNA‐binding proteins, which play critical roles in mtRNA processing, maturation, and mitochondrial ribosome assembly [28, 29, 30]. Previous studies have demonstrated that cytoplasmic RNA G4s contribute to phase‐separated assemblies and RNA granule formation [31, 32]. We therefore hypothesized that mtRNA G4s may also participate in MRG formation. Consistent with this hypothesis, our data revealed prominent colocalization between **MitoQUMA**‐labeled mtRNA G4s and GRSF1 (Figure 2G). To further probe the relationship between mtRNA G4s and MRGs, we overexpressed the canonical MRG marker, FASTKD2, which does not alter mtRNA G4 abundance (Figure S7). Under super‐resolution structured illumination microscopy, we observed significant colocalization between **MitoQUMA** signals and FASTKD2‐labeled granules (Figure 3A). Moreover, live‐cell real‐time imaging revealed correlated trajectories (with PCC values around 0.8) and dynamic fusion‐fission events between mtRNA G4s and FASTKD2‐indicated MRGs (Figure 3B), suggesting that mtRNA G4s are embedded within MRGs and may play a role in their assembly through phase separation.

To investigate the functional role of mtRNA G4s in MRG assembly, we sought a ligand capable of selectively modulating these structures. Although no such small molecules have been reported, we noted that the prototype compound QUMA‐1 can act as a small‐molecule ligand to study the relationship between cytoplasmic RNA G4s and RNA granule formation [33, 34]. This observation suggests that **MitoQUMA** may have similar potential. Therefore, we treated cells with increasing concentrations of **MitoQUMA** and observed that, at the imaging working concentration (2 µm), **MitoQUMA** had little effect on FASTKD2 foci. However, at higher concentrations (20 and 50 µm), the number of FASTKD2 foci significantly decreased (Figure 3C), while the levels of housekeeping proteins such as Actin and Histone H3 remained largely unchanged (Figure S8). Consistently, siRNA‐mediated knockdown of FASTKD2 had minimal impact on **MitoQUMA** foci (Figure S9). By comparison, knockdown of GRSF1, which is known to promote mtRNA G4 formation (Figure 2H), led to a significant reduction in MRG numbers (Figure 3D). Together, these results indicate that mtRNA G4 formation may hinder MRG assembly.

Interestingly, unlike cytoplasmic RNA G4s that often serve as scaffolds to promote phase separation, mitochondrial RNA G4s appear to act as brakes on MRG formation. We speculated that this phenomenon might be related to the unique function of MRGs. MRGs are enriched in nascent mtRNAs and harbor RNA‐processing machinery, forming essential hubs for mtRNA maturation [27, 28, 29, 30, 35]. The assembly of MRGs is tightly coupled to efficient mtRNA processing. Premature or excessive G4 folding may create a structural barrier that hinders the proper association of RNA‐binding proteins and the subsequent processing of mtRNAs. When processing is compromised, mtRNA maturation is impaired, rendering MRGs unstable and prone to disassembly. It is precisely for this reason that GRSF1, an essential component of MRGs and a key regulator of mtRNA processing, functions to unwind mtRNA G4s, thereby preventing the aberrant accumulation of G‐rich mitochondrial L‐strand transcripts and maintaining proper processing of their complementary coding counterparts [9, 27, 35]. Consistent with this idea, high‐dose **MitoQUMA** treatment led to a marked accumulation of mitochondrial L‐strand transcripts such as lncCYTB and lncND5 (Figure 3E), accompanied by a widespread downregulation of mRNAs encoding the 13 core mitochondrial respiratory chain proteins (Figure 3F).

### 2.4 A Chemical Genetic Screen Identifies the Wnt/β‐Catenin–GRSF1–mtRNA G4 Axis Regulating MRG Assembly in Cancer Cells

How cells control mtRNA G4 folding and MRG assembly, and the biological consequences of this regulation, remain largely unknown. Addressing this question is critical for elucidating the functional roles of mtRNA G4s in mitochondrial gene expression. To this end, we leveraged **MitoQUMA** as a live‐cell imaging tool and designed a high‐content chemical genetic screening approach in HeLa cells to identify signaling pathways that modulate mtRNA G4 formation and subsequent MRG dynamics [36, 37, 38]. Specifically, we screened a library containing 167 inhibitors targeting pathways known to influence mitochondrial function and quantified their effects on mtRNA G4 abundance. Given that some inhibitors might perturb mitochondrial membrane potential or alter mtDNA copy number, thereby indirectly affecting **MitoQUMA** fluorescence, we included our previously validated mitochondrial nucleic acid probe MitoTO as an internal control. By comparing fluorescence changes between **MitoQUMA** and MitoTO, we aimed to identify compounds that selectively modulate mtRNA G4 folding without broadly affecting mitochondrial nucleic acids. Such compounds likely point to signaling pathways directly regulating MRG assembly via mtRNA G4s rather than causing nonspecific mitochondrial stress.

**FIGURE 4:**
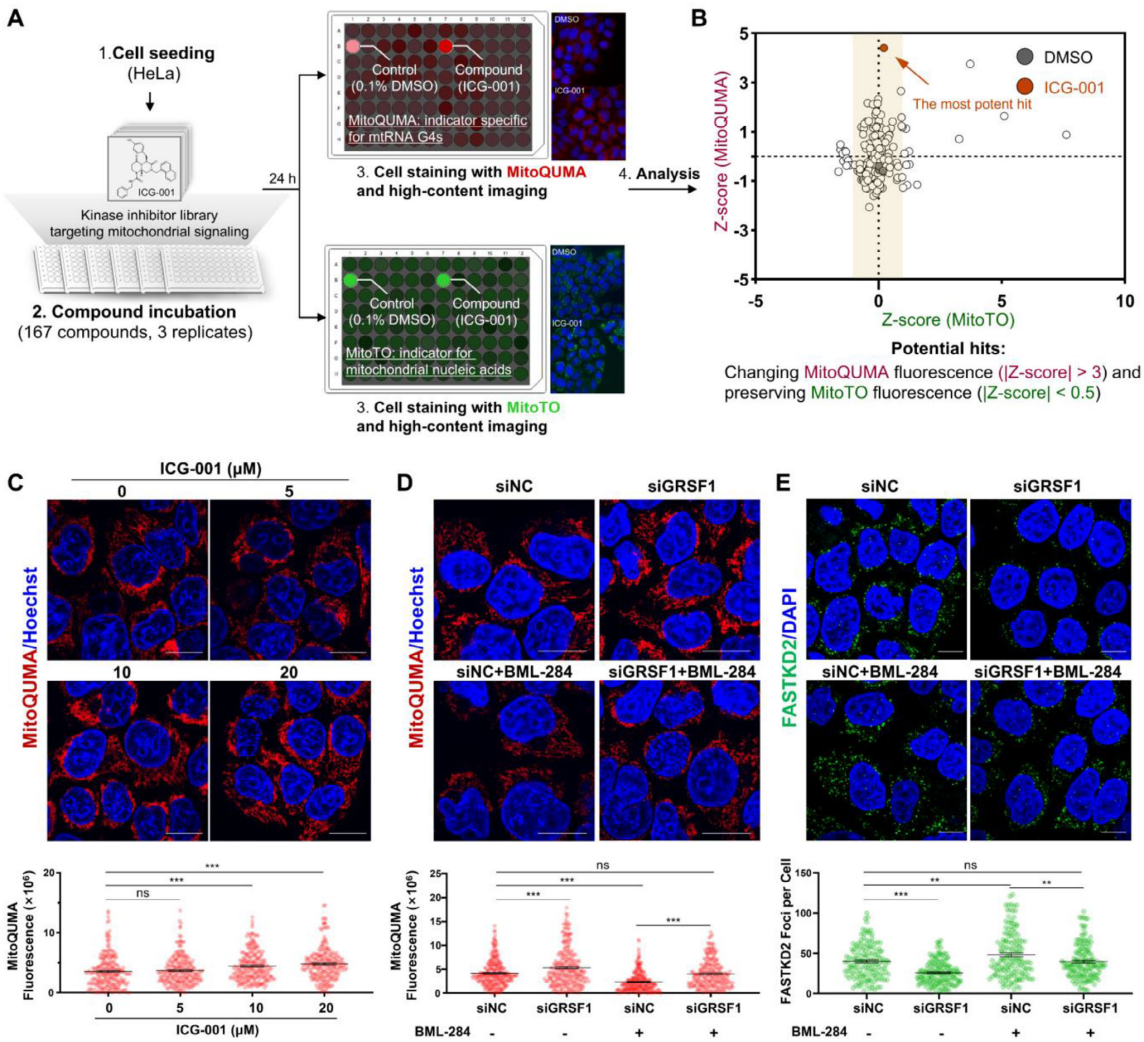
A chemical genetic screen identifies the Wnt/β‐Catenin‐GRSF1‐mtRNA G4 axis regulating MRG assembly in cancer cells. (A) Depiction of the screening workflow. The data obtained regarding the fluorescence changes of **MitoQUMA** and MitoTO were standardized by calculating the *Z‐*score for each inhibitor. (B) *Z*‐score value for each inhibitor was plotted to identify hits with thresholds of |*Z*‐score_(_ **_MitoQUMA_**_)_ |>3 and |Z‐score_(MitoTO)_ | <0.5. (C) Live HeLa cells were treated with varying concentrations of ICG‐001, followed by staining with 2 µm **MitoQUMA**. (D) Live HeLa cells were transfected with siRNA to knock down GRSF1 expression and then treated with or without 20 nm BML‐284, followed by staining with 2 µm **MitoQUMA**. (E) Influence of MRG formation in HeLa cells upon siGRSF1 treatment with or without subsequent 20 nM BML‐284 exposure. MRGs were indicated by immunofluorescence staining of FASTKD2. For each sample of cell image, approximately 100 cells were measured. Biological replicates (*n* = 3) were taken. The data are presented as mean ± SEM, and statistical significance is determined by the one‐way ANOVA with Dunnett's multiple comparisons test as (ns) not significant, (^*^) *p* < 0.05, (^**^) *p* < 0.01, and (^***^) *p* < 0.001. Scale bars for cell image: 10 µm.

The detailed screening workflow is illustrated in Figure 4A. Briefly, HeLa cells were seeded into 96‐well plates and treated with each compound, using 0.1% DMSO as the control. After 24 h, cells were stained with **MitoQUMA** (red) and MitoTO (green) respectively, with nuclei stained by Hoechst 33342 (blue). High‐content imaging was then used to quantify fluorescence intensity changes relative to controls, followed by Z‐score normalization (Data S1). We defined |*Z*‐score _(_ **_MitoQUMA_**_)_| >3 and |*Z*‐score _(MitoTO)_| <0.5 as thresholds for compounds that selectively modulate mtRNA G4s without broadly affecting mitochondrial nucleic acids. As shown in Figure 4B, only the Wnt/β‐Catenin pathway inhibitor ICG‐001 met this criterion, inducing the most pronounced increase in **MitoQUMA** fluorescence (*Z*‐score = 4.403) while having minimal effect on MitoTO (*Z*‐score = 0.214). These findings indicate that Wnt/β‐Catenin signaling may serve as a regulatory pathway influencing mtRNA G4 abundance.

To validate this pathway's role, we treated HeLa cells with increasing doses of ICG‐001 and observed a dose‐dependent increase in mtRNA G4 number as indicated by **MitoQUMA**, with no appreciable effect on MitoTO fluorescence (Figure 4C; Figure S10A). Conversely, treatment with the Wnt/β‐Catenin agonist BML‐284 suppressed **MitoQUMA** signal (Figure S10B). These results demonstrate that Wnt/β‐catenin signaling dynamically regulates mtRNA G4 formation. Given that Wnt activation modulates a broad spectrum of downstream genes, including cell cycle regulators, transcription factors, migration proteins, and metabolic enzymes, we next focused on GRSF1, the only known Wnt downstream target directly implicated in mtRNA metabolism [39]. We found that ICG‐001 markedly decreased, whereas BML‐284 increased GRSF1 mRNA and protein levels (Figure S10C,D). Moreover, BML‐284 treatment rescued both mtRNA G4 accumulation and MRG reduction induced by GRSF1 knockdown (Figure 4D,E). These results demonstrate that Wnt/β‐catenin signaling regulates mtRNA G4 folding and MRG assembly through GRSF1, thereby highlighting a Wnt/β‐catenin–GRSF1–mtRNA G4 axis as a key regulatory pathway in MRG assembly.

### 2.5 Enhanced mtRNA G4 Formation Disrupts MRG Assembly, Impairing Mitochondrial Respiration and Arresting Cell Proliferation

**FIGURE 5:**
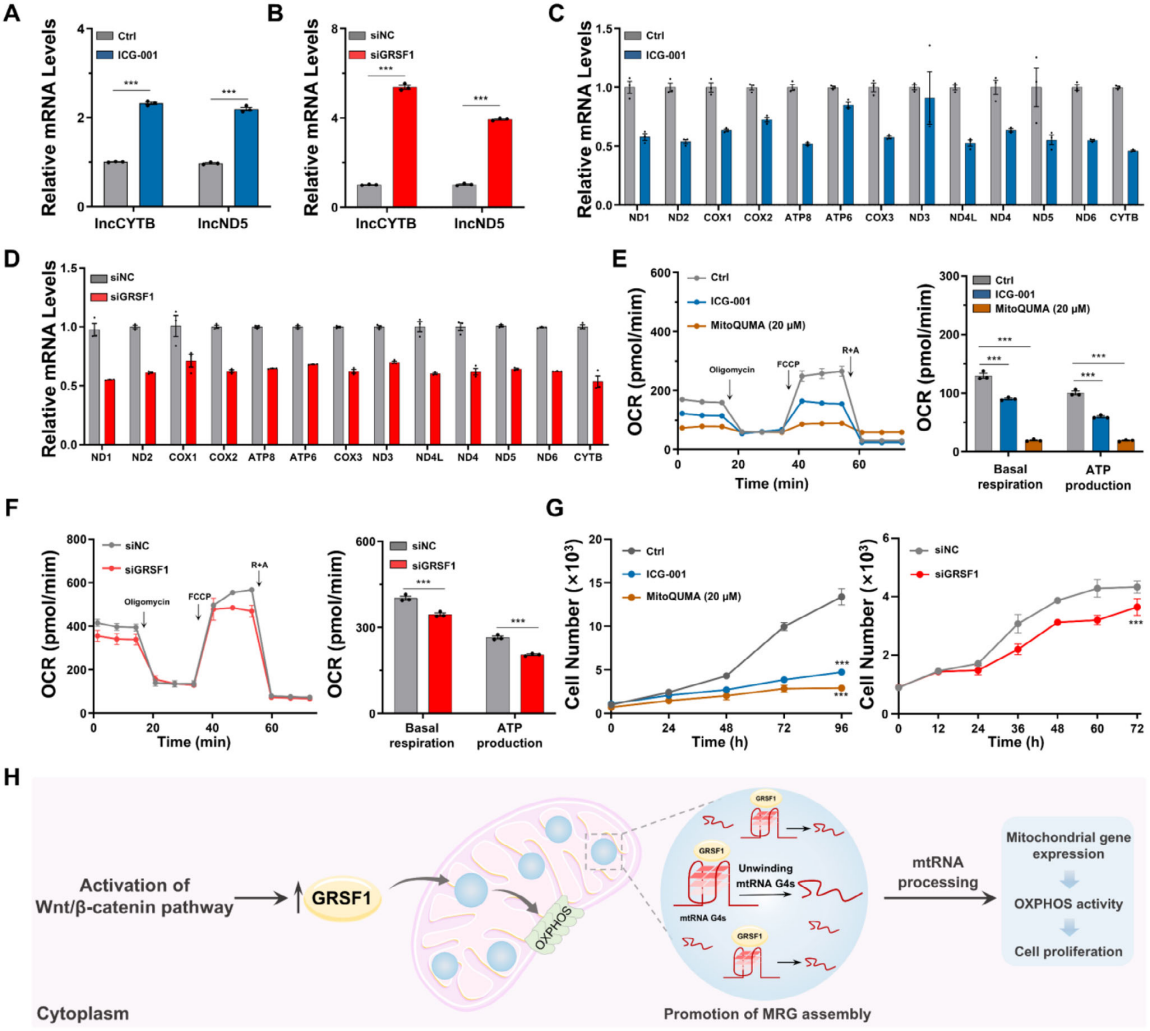
Enhanced mtRNA G4 formation disrupts MRG assembly, impairing mitochondrial respiration and arrests cell proliferation. (A‐B) L‐strand transcripts (lncCYTB and lncND5) expression levels in HeLa cells following 24 h treatment with 10 µm ICG‐001 (A) or siRNA‐mediated GRSF1 knockdown (B). (C,D) Mitochondrial gene expression levels in HeLa cells following 24 h treatment with 10 µm ICG‐001 (C) or siRNA‐mediated GRSF1 knockdown (D). (E,F) Effects of 24 h treatment with 10 µm ICG‐001 or high‐dose 20 µm MitoQUMA (E) and 24 h siRNA‐mediated silencing of GRSF1 (F) on oxygen consumption rate in HeLa cells. (G) Effects of ICG‐001 (10 µm), high‐dose MitoQUMA (20 µm) treatment, and GRSF1 silencing on cell viability in HeLa cells. (H) The Wnt/β–Catenin–GRSF1 axis modulates mtRNA G4 folding and MRG assembly, thereby maintain mtRNA processing and mitochondrial gene expression, OXPHOS activity, and ultimately cancer cell proliferation. Biological replicates (*n* = 3) were taken. The data are presented as mean ± SEM, and statistical significance is determined by the two‐way ANOVA with Sidak's multiple comparisons test as (ns) not significant, (^*^) *p* < 0.05, (^**^) *p* < 0.01, and (^***^) *p* < 0.001. Scale bars for cell image: 10 µm.

Building on the above findings, we next examined the consequences of Wnt/β‐catenin–GRSF1–mtRNA G4 axis modulation on mtRNA homeostasis and cellular function. We observed that treatment with ICG‐001, which increases mtRNA G4 abundance and reduces MRG number through Wnt/β‐catenin inhibition, and GRSF1 knockdown, which exerts similar effects, both led to abnormal accumulation of mitochondrial L‐strand transcripts, including lncCYTB and lncND5 (Figure 5A,B). Correspondingly, mRNAs encoding 13 key mitochondrial respiratory chain proteins were significantly downregulated (Figure 5C,D), consistent with the observations following high‐dose **MitoQUMA** treatment (Figure 3E,F).

As a consequence of this mitochondrial gene expression inhibition, ICG‐001 or high‐dose **MitoQUMA** treatment led to a marked decrease in cellular oxygen consumption, reflected by reductions in both basal respiration and ATP production, consistent with impaired OXPHOS (Figure 5E). Similar results were observed in GRSF1‐knockdown cells (Figure 5F), demonstrating that mtRNA G4 accumulation and the consequent reduction of MRGs profoundly impair mitochondrial energy metabolism. Consistently, ICG‐001 treatment, high‐dose **MitoQUMA** treatment, and GRSF1 knockdown each led to varying degrees of suppression of HeLa cell proliferation (Figure 5G). Notably, Wnt/β‐catenin signaling is critical for cellular stress adaptation. Prior studies have shown that under hypoxic conditions, activation of the Wnt/β‐catenin pathway promotes survival and proliferation of tumor cells [40]. In line with this, our previous work revealed a positive correlation between MRG abundance and cancer cell proliferation under hypoxia or cold stress [41]. Building on these observations, the present study further connects these results by defining the Wnt/β‐catenin–GRSF1–mtRNA G4 axis as a previously unrecognized regulatory pathway controlling MRG assembly, mitochondrial gene expression, OXPHOS activity, and ultimately cancer cell proliferation (Figure 5H).

## 3 Conclusion

Mitochondria have their own genome and transcription‐translation system that function independently of the nucleus. Although the human mitochondrial genome has undergone substantial evolutionary streamlining, it retains numerous G‐rich sequences capable of forming G4 structures, underscoring their potential importance for mitochondrial function. Among all classes of G4s, mtRNA G4s remain the least studied, and their biological roles are poorly understood, mainly because no suitable intracellular probes are available. In this study, building on our previously developed and commercialized cytoplasmic RNA G4 probe QUMA‐1, we used rational structural modifications combined with imaging‐based screening to establish structure‐activity relationships and ultimately developed **MitoQUMA**, which, to our knowledge, represents the first fluorescent probe enabling visualization of mtRNA G4s in live cells.

Among the newly synthesized QUMA‐1 derivatives, **MitoQUMA** is the compound that is most structurally similar to QUMA‐1. In solution, **MitoQUMA** exhibits comparable fluorescence responses toward mtRNA G4s and mtDNA G4s. However, in cellular contexts, **MitoQUMA** can be used to track mtRNA G4s. This behavior is similar to our previous observations with QUMA‐1, which showed limited discrimination between RNA and DNA G4s in cell‐free assays but preferentially visualized cytoplasmic RNA G4s in live cells. Based on these observations, we propose that differences in the microenvironments occupied by mtRNA and mtDNA within mitochondria may contribute to the observed cellular behavior of **MitoQUMA**. Within mitochondria, nascent mtRNAs, including G‐rich transcripts such as mitochondrial long noncoding RNAs, are present at high local concentrations and can undergo phase separation with associated proteins to form compartmentalized MRGs. In contrast, mtDNA is organized within nucleoid structures that constitute a distinct and more compact microenvironment [42]. The unique environment within MRGs, which may feature locally enriched, dynamic, and more accessible mtRNA G4s, as well as higher‐order assemblies arising from interactions among multiple mtRNA G4s, could therefore favor the engagement of **MitoQUMA**. Consistent with this notion, using **MitoQUMA**, we observed a close association between mtRNA G4 formation and the assembly of MRGs.

Interestingly, previous studies have shown that cytoplasmic RNA G4s often act as scaffolds that facilitate RNA‐protein phase separation and promote the assembly of RNA granules, such as stress granules (SGs) [31, 32]. By contrast, in mitochondria, mtRNA G4 formation hinders rather than promotes MRG assembly, and efficient MRG formation requires a reduction in mtRNA G4 abundance. We propose that this discrepancy reflects distinct biological functions: SGs are transient, stress‐induced protective structures in which stable G4s promote their rapid assembly. By comparison, MRGs serve as constitutive platforms for nascent mtRNA processing, maturation, and ribosome assembly, where premature or excessive G4 folding may create a structural barrier that hinders the proper association of RNA‐binding proteins and subsequent mtRNA processing. When RNA processing is compromised, mtRNA maturation is impaired, and MRG becomes unstable and undergoes disassembly [28, 29, 43]. These findings highlight the context‐dependent complexity of RNA G4 function.

To further elucidate how mtRNA G4‐dependent regulation of MRG assembly impacts mitochondrial bioenergetics and cellular function, we established a chemical genetic screening platform based on **MitoQUMA**. Through this approach, we identified the Wnt/β‐catenin–GRSF1–mtRNA G4 axis as a previously unrecognized regulatory pathway that controls MRG assembly, thereby influencing mitochondrial gene expression, OXPHOS activity, and ultimately cancer cell proliferation. Whereas most prior studies have focused on the role of Wnt/β‐catenin signaling in metabolic reprogramming through nuclear targets (e.g., c‐Myc, HIF‐1α, PDK) [44, 45], our findings reveal a distinct mechanism whereby Wnt signaling modulates mitochondrial RNA secondary structures and phase separation, thereby directly affecting mitochondrial gene regulation and OXPHOS activity. This work provides additional insight into how signaling pathways may regulate mitochondrial RNA biology and points to possible implications for targeting cancer metabolism.

The results obtained using **MitoQUMA** are internally consistent across experiments and support its use under the conditions examined. At the same time, we recognize that fluorogenic probes are inherently influenced by probe concentration and imaging conditions, which defines the practical boundaries of intensity‐based approaches. In this regard, recent advances in ratiometric fluorescent probes and fluorescence lifetime‐based probe strategies provide promising directions for overcoming some of these limitations [46, 47]. Within the scope of the present study, we therefore combined multiple complementary controls, independent perturbations, bidirectional regulatory strategies, and quantitative analyses across large cell populations to reduce potential bias and strengthen the interpretability of our observations. These considerations also highlight that future applications of **MitoQUMA** should similarly rely on integrated experimental designs and on converging trends observed across different conditions rather than on any single measurement.

Taken together, building upon QUMA‐1, **MitoQUMA** extends this chemical scaffold to enable live‐cell study of mtRNA G4s within mitochondria. We hope that much like QUMA‐1 has facilitated progress in the RNA G4 field, **MitoQUMA** may similarly contribute to advancing investigation of mtRNA G4s, helping to enable a more systematic understanding of how RNA secondary structures influence mitochondrial gene expression, phase separation dynamics, and organelle homeostasis. At the same time, we recognize that defining the scope and limitations of this probe requires continued exploration, and that future advances may further refine this area. In this context, we view **MitoQUMA** as a useful chemical tool and an initial step toward systematic investigation of mtRNA G4‐associated processes.

## 4 Experimental Section

### 4.1 Compounds, Oligonucleotides, and Other Meterials

All oligonucleotide sequences used in this study are provided in Tables S1 and S2. Detailed synthesis and characterization procedures of Q1‐Q4, Q5 (MitoQUMA) and TPP‐QUMA, biophysical methods and more detailed materials are described in the Supporting Information.

### 4.2 Fluorescence Spectroscopy

Fluorescence studies were carried out on a Fluoromax‐4 Spectrofluorometer (HORIBA). The emission spectra of **MitoQUMA** were acquired by exciting the sample at 555 nm. The emission spectra collection range of **MitoQUMA** was 575–800 nm. All oligonucleotides (Sangon Biotech, Table S1) were preannealed by heating at 95°C for 5 min, followed by slow cooling to room temperature in Tris‐HCl buffer (10 mm, pH 7.4) with 100 mm KCl. Small aliquots of a stock solution of oligonucleotides were added into the solution containing **MitoQUMA** at a fixed concentration (1 µm). For the titration studies, the final concentration of oligonucleotides was varied from 0 to 3 µm. After the addition of each sample, the mixture was stirred and allowed to equilibrate for at least 2 min.

### 4.3 Cell Lines and Cell Cultures

The human cervical HeLa cell lines was obtained from the cell bank of Sun Yat‐Sen University Experimental Animal Center (China). HeLa cells were cultured in MEM (Procell, PM150410) supplemented with 10% fetal bovine serum (ExCell Bio, FSP500). All cells are cultured at 37°C in an incubator with a humidified atmosphere of 20% O_2_ and 5% CO_2_. For the preparation of ρ^0^ HeLa cells, parental HeLa cells were cultured for 30–40 days in low‐dose EtBr (Sigma) (50 ng/mL) supplemented with 1 mm pyruvate (Sigma) and 50 mg/mL uridine (Sigma) followed by transfer to medium lacking EtBr.

### 4.4 Live Cell and Fixed Cell Imaging

Cells were seeded in a glass‐bottom 96‐well plate and grew overnight. The cells were then stained with probes in MEM in a 5%CO_2_ atmosphere at 37°C for 3 h. Hoechst33342 or DAPI was used to indicate the nucleus. For live cell imaging, all treated cells were stained with 2 µm **MitoQUMA** or 2 µm MitoTO for 3 h before imaging, and 1 µm Pyronin Y (TargetMol, T16698) for 1 h before imaging. The emission spectra of **MitoQUMA** and MitoTO were acquired by exciting samples at 561 and 488 nm, respectively. The emission spectra collection range of **MitoQUMA** and MitoTO were 580–800 and 500–700 nm, respectively. Pyronin Y was imaged under the same excitation and emission settings as **MitoQUMA**. MitoTracker Green (Invitrogen, M7514) and MitoTracker Deep Red (Invitrogen, M22426) were used to indicate mitochondria, PicoGreen (YEASEN, 12641ES01) was used to indicate mtDNA. The treatment concentrations of IMT1 (TargetMol, T8841), Chloramphenicol (TargetMol, T1205), RHPS4 (TargetMol, T6967), Valinomycin (TargetMol, TP1072), Ionomycin (Beyotime, S1672), ICG‐001 (TargetMol, T6113) and BML‐284 (TargetMol, T8820) have been provided in the figure legend. For valinomycin (10 µm together with 200 mm KCl) or ionomycin (20 µm together with 20 mm KCl) treatment, cells were first stained with the fluorescent probes, and were then treated with valinomycin for 10 min or ionomycin for 20 min. For fixed cell imaging, the cells were fixed with 4% paraformaldehyde in DEPC‐PBS at room temperature for 15 min and permeabilized with 0.5% Triton X‐100 in DEPC‐PBS at 37°C for 20 min. Then, cells were incubated with 200 units mL^−1^ RNase A (Thermo Fisher Scientific, EN0531) or DNase I (Thermo Fisher Scientific, EN0523) for 5 h after permeabilization. Imaging was carried out on an FV3000 laser scanning confocal microscope (Olympus) with a 60 × objective lens (for confocal imaging) or a HIS‐SIM imaging system (Computational SR) with a 100 × 1.5 Apo oil‐immersion objective (for super‐resolution imaging). The images were analyzed with Imaris software (Bitplane Corp.).

### 4.5 Chemical Genetics Screening and Data Analysis

The kinase inhibitor library containing 167 compounds was purchased from TargetMol. For screening in 96‐well plates, a seeding density of 5000 cells per well for HeLa cells was chosen. On the second day, the cells were exposed to kinase inhibitors (10 µm) or 0.1% DMSO. After incubation for 24 h, the cells were stained with either **MitoQUMA** (2 µm, 3 h) or MitoTO (2 µm, 3 h). IamgeXpress Micro Confocal (Molecular Devices) high‐content screening station was used to quantify the fluorescence signal in cells. The high‐content analysis automatically focused on the fluorescence channel of Hoechst33342 and captured the channel of either **MitoQUMA** or MitoTO. The mean fluorescence signal of **MitoQUMA** and MitoTO per cell was analyzed. The initial fluorescence signal of **MitoQUMA** or MitoTO in each well was first recorded as F_0_. After treating inhibitors, the final fluorescence signal of **MitoQUMA** or MitoTO was recorded as F. The fluorescence intensity change was calculated by the formulation of F/F_0_. Positive hits for each compound were identified as follows. *Z*‐scores of the mean fluorescence intensity changes in **MitoQUMA** or MitoTO were calculated for each compound when compared with the negative control. Subsequently, these data were further filtered to identify compounds that reproducibly have a |*Z*‐score (**MitoQUMA**)| >3 and |*Z*‐score (MitoTO)| <0.5. Screening data is summarized in the Data S1 file. Data post‐processing was conducted using Prism scripts.

### 4.6 Oxygen Consumption Rate Assays

The oxygen consumption rate (OCR) was measured using Seahorse XF Cell Mito Stress Kit. Briefly, cells were seeded into a Seahorse XF96 cell culture microplate (8 × 10^3^ cells/well) and allowed to adhere overnight, at which time the cell number for each group was very similar. The cells were then infected or transfected as described above. After 24 h treatment, the cells were used for measurement of OCR. OCR was monitored at baseline and throughout sequential injections of oligomycin, FCCP and rotenone/antimycin. Data was analyzed by Seahorse XF96 Wave software. The results were normalized to cell numbers.

### 4.7 Statistical Analysis

Data are expressed as mean ± SEM. Pearson's correlation coefficient (PCC) used ImageJ. Other analyses used GraphPad Prism 9.0. Statistical comparisons between groups were performed using two‐sided Student's unpaired *t*‐test for two groups, one‐way and two‐way analysis of variance (ANOVA) for comparisons involving three or more groups.

## Conflicts of Interest

The authors declare no conflicts of interest.

## Supporting information




**Supporting File 1**: advs74416‐sup‐0001‐SuppMat.pdf.

**Supporting File 2**: advs74416‐sup‐0002‐DataFile.zip.

## Data Availability

The data that support the findings of this study are available from the corresponding author upon reasonable request.
