# Supplementary material for: Illuminating Mitochondrial RNA G‐Quadruplexes as Structural Brakes on RNA Granule Assembly and OXPHOS
Source: Adv Sci (Weinh). 2026 Feb 12;13(23):e23462. doi: 10.1002/advs.202523462 (PMC13104114; doi:10.1002/advs.202523462)
Supplement: Supplementary file 1 — Supporting File 1: advs74416‐sup‐0001‐SuppMat.pdf. [file ADVS-13-e23462-s002.pdf]

Supporting Information

Illuminating Mitochondrial RNA G-Quadruplexes as Structural Brakes on RNA Granule Assembly and OXPHOS

Gui-Xue Tang,+[1, 2] Jia-Tong Yan,+[2] Mao-Lin Li,+[2] Cui Zhou,[2] Jian Wang,[2] Shuo-Bin Chen,*[2] Zhi-Shu Huang,[1, 2] and Jia-Heng Tan*[1, 2]

[1] State Key Laboratory of Oncology in South China, Sun Yat-sen University Cancer Center, Guangzhou, China.

[2] School of Pharmaceutical Sciences, Sun Yat-sen University, Guangzhou, China.

+ These authors contributed equally to this work

* Correspondence: chenshb8@mail.sysu.edu.cn (Shuo-Bin Chen), tanjiah@mail.sysu.edu.cn (Jia-Heng Tan)

Table of Content

1. Supplementary Methods S2
1.1 Synthesis and Characterization S2
Scheme S1. Synthesis of **Q1-Q4, Q5 (MitoQUMA) and TPP-QUMA** S2
1.2 Circular Dichroism Spectroscopy S5
1.3 Cytotoxicity and Cell Number Analysis S5
1.4 Plasmid Constructions and siRNA S5
1.5 Immunofluorescence S6
1.6 RNA Extraction and Real-time PCR S6
1.7 Western Blot S7

2. Supplementary Materials S8
Table S1. Sequence of oligomers used in the study S8
Table S2. PCR primers used in the present study S8

3. Other Supporting Spectra and Graphs S9
Figure S1. Super-resolution and confocal imaging of **MitoQUMA** S9
Figure S2. The CD spectra of mtRNA and mtDNA G4s S9
Figure S3. Photostability and cytotoxicity of **MitoQUMA** S10
Figure S4. Live HeLa cells were stained with varying concentrations and times of **MitoQUMA** S10
Figure S5. The CD melting curves of mtRNA and mtDNA G4s with or without **MitoQUMA** S11
Figure S6. Validation of selectivity of **MitoQUMA** S12
Figure S7. The impact of overexpressed FASTKD2 on the formation of mtRNA G4s S13
Figure S8. The impact of high concentration of **MitoQUMA** on housekeeping proteins S13
Figure S9. The impact of FASTKD2 knockdown on the formation of mtRNA G4s S14
Figure S10. The Wnt/β-catenin-GRSF1 signaling axis regulates MRG dynamics S15
NMR, HRMS and HPLC spectra of the final compounds S16

4. Reference S20

1. Supplementary Methods

1.1 Synthesis and Characterization

^1H and ^{13}C NMR spectra were recorded on a Bruker Ascend™ 400 or Ascend™ 500 spectrometer. High-resolution mass spectra (HRMS) were recorded on a Shimadzu LCMS-IT-TOF. Flash column chromatography was performed with silica gel (200-300 mesh) purchased from Qingdao Haiyang Chemical Co. Ltd. The purity of synthesized compound was confirmed to be higher than 95% by using analytical HPLC performed with a dual pump Shimadzu LC-20 AB system equipped with a Ultimate XB-C18 column (4.6 × 250 mm, 5 μm) and eluted with a mixture of methanol/water (20%—60%) containing 0.1% TFA at a flow rate of 0.5 mL/min. All chemical reagents and solvents for synthesis and characterization were purchased from commercial suppliers (J&K Scientific Ltd., Sigma-Aldrich Chemical Co. and Shanghai Aladdin Bio-Chem Technology Co. Ltd.) unless otherwise specified and were used without further purification. The intermediate **1** and final compounds **Q1-Q4** were synthesized following published procedures[1,2].

Scheme S1. Synthesis of final compounds **Q1-Q4**, **Q5 (MitoQUMA)** and **TPP-QUMA**.[a]

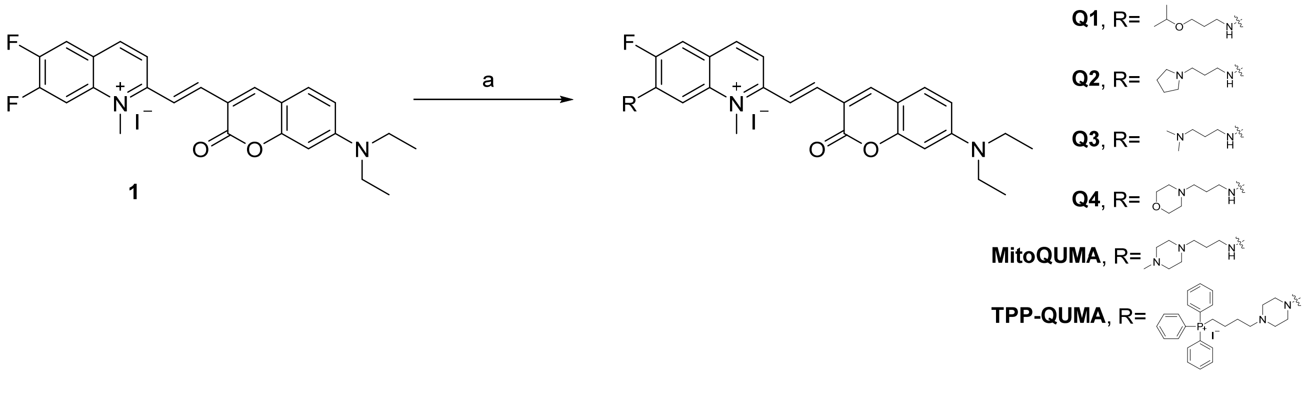

[a] **Reagent and conditions**: (a) K_2CO_3, amine chain, acetonitrile, room temperature, 4 h.

General procedure to Q1-Q4, Q5 (MitoQUMA) and TPP-QUMA

To a solution of compound **1** (280 mg, 0.5 mmol) in acetonitrile (2.0 mL), amino chain (1.0 mmol) and a catalytic amount of K_2CO_3 was added. The reaction mixture was stirred at room temperature for 4 h. After completion, CH_2Cl_2 (30 mL) and H_2O (15 mL) were added, and the organic layer was extracted and concentrated under reduced pressure. The crude product was purified by using flash column chromatography with MeOH/CH_2Cl_2 (20:1) containing 1% $NH_3 \cdot H_2O$ elution to afford a brownish black solid as final compound **Q1-Q4**, **Q5** (**MitoQUMA**) and **TPP-QUMA**.

Q1, yield 69%. ^{1}H NMR (400 MHz, CD_3OD) δ 8.36 (d, *J* = 8.5 Hz, 1H), 8.10 (s, 1H), 7.98 (d, *J* = 15.6 Hz, 1H), 7.82 (d, *J* = 8.5 Hz, 1H), 7.65 – 7.52 (m, 2H), 7.41 (d, *J* = 8.9 Hz, 1H), 6.93 (d, *J* = 7.0 Hz, 1H), 6.72 (dd, *J* = 8.9, 2.4 Hz, 1H), 6.36 (d, *J* = 2.4, 1H), 4.26 (s, 3H), 3.72 – 3.63 (m, 3H), 3.54 (t, *J* = 6.8 Hz, 2H), 3.48 (q, *J* = 7.2 Hz, 4H), 2.02 (p, *J* = 6.8 Hz, 2H), 1.28 – 1.12 (m, 12H). ^{19}F NMR (375 MHz, CD_3OD) δ -130.2. ^{13}C NMR (125 MHz, DMSO-*d_6*) δ 159.3, 156.0, 153.0 (d, *J* = 1.7 Hz), 151.9, 150.9 (d, *J* = 252.5 Hz), 144.8, 143.8 (d, *J* = 15.4 Hz), 141.2 (d, *J* = 3.9 Hz), 139.5, 138.2, 130.3, 120.1 (d, *J* = 10.7 Hz), 118.2, 115.1, 113.2, 111.9 (d, *J* = 20.0 Hz), 109.8, 108.1, 96.0, 94.4 (d, *J* = 3.9 Hz), 70.3, 65.0, 44.0 (2C), 40.1, 39.1, 27.9, 21.7 (2C), 12.0 (2C). HRMS (ESI) m/z: calcd for $C_{31}H_{37}FN_3O_3^+$ $[M-I]^+$ 518.2813, found 518.2814. HPLC purity: 96.5%.

Q2, yield 73%. ^{1}H NMR (400 MHz, CD_3OD) δ 8.44 (d, *J* = 8.5 Hz, 1H), 8.14 (s,1H), 8.05 (d, *J* = 15.4 Hz, 1H), 7.87 (d, *J* = 8.6 Hz, 1H), 7.68 (d, *J* = 11.1 Hz, 1H), 7.61(d, *J* = 15.4 Hz, 1H), 7.46 (d, *J* = 9.0 Hz, 1H), 7.06 (d, *J* = 7.0 Hz, 1H), 6.77 (dd, J = 9.0, 2.4 Hz, 1H), 6.45 (d, *J* = 2.4 Hz, 1H), 4.35 (s, 3H), 3.64 (t, *J* = 6.7 Hz, 2H), 3.51(q, *J* = 7.0 Hz, 4H), 3.44 – 3.34 (m, 6H), 2.24 (p, *J* = 6.7 Hz, 2H), 2.16 – 2.06 (m, 4H),1.24 (t, *J* = 7.0 Hz, 6H). ^{19}F NMR (375 MHz, CD_3OD) δ -129.5. ^{13}C NMR (100 MHz,DMSO-*d_6*) δ 159.3, 156.0, 153.0 (d, *J* = 1.7 Hz), 151.9, 150.9 (d, *J* = 254.6 Hz), 144.9,143.7 (d, *J* = 15.0 Hz), 141.2 (d, *J* = 4.0 Hz), 139.5, 138.2, 130.3, 120.1 (d, *J* = 9.1 Hz),118.1, 115.1, 113.2, 111.9 (d, *J* = 20.1 Hz), 109.8, 108.1, 96.0, 94.8 (d, *J* = 3.7 Hz),52.8 (2C), 52.0, 44.0 (2C), 40.3, 38.6, 24.4, 22.5 (2C), 12.0 (2C). HRMS (ESI) m/z: calcd for $C_{32}H_{38}FN_4O_2^+$ $[M-I]^+$ 529.2973, found 529.2974. HPLC purity: 95.2%.

Q3, yield 56%. ^{1}H NMR (500 MHz, CD_3OD) δ 8.48 (d, *J* = 8.5 Hz, 1H), 8.18 (s, 1H), 8.12 (d, *J* = 15.5 Hz, 1H), 7.90 (d, *J* = 8.6 Hz, 1H), 7.73 (d, *J* = 11.0 Hz, 1H), 7.65 (d, *J* = 15.5 Hz, 1H), 7.49 (d, *J* = 9.0 Hz, 1H), 7.10 (d, *J* = 7.1 Hz, 1H), 6.80 (dd, *J* = 9.0, 2.4 Hz, 1H), 6.51 (d, *J* = 2.4 Hz, 1H), 4.38 (s, 3H), 3.64 (t, *J* = 6.9 Hz, 2H), 3.53 (q, *J* = 7.1 Hz, 4H), 3.30 (t, *J* = 6.9 Hz, 2H), 2.91 (s, 6H), 2.22 (p, *J* = 6.9 Hz, 2H), 1.25 (t, *J* = 7.1 Hz, 6H). ^{19}F NMR (470 MHz, CD_3OD) δ -129.7. ^{13}C NMR (125 MHz, DMSO-*d_6)* δ 159.6, 156.2, 153.4 (d, *J* = 1.8 Hz), 152.1, 151.2 (d, *J* = 253.6 Hz), 145.1, 144.2 (d, *J* = 15.0 Hz), 141.5 (d, *J* = 5.2 Hz), 139.9, 138.4, 130.5, 120.4 (d, *J* = 9.6 Hz), 118.7, 115.4, 113.5, 112.1 (d, *J* = 20.1 Hz), 110.1, 108.3, 96.3, 94.7 (d, *J* = 3.4 Hz), 56.4, 44.4 (2C), 44.2 (2C), 41.1, 40.2, 24.9, 12.2 (2C). HRMS (ESI) m/z: calcd for $C_{30}H_{36}FN_4O_2^+$ $[M-I]^+$ 503.2817, found 503.2817. HPLC purity: 96.0%.

Q4, yield 68%. ^{1}H NMR (400 MHz, CD_3OD) δ 8.49 (d, *J* = 8.5 Hz, 1H), 8.18 (s, 1H), 8.16 (d, *J* = 15.5 Hz, 1H), 7.87 (d, *J* = 8.5 Hz, 1H), 7.75 (d, *J* = 11.2 Hz, 1H), 7.65 (d, *J* = 15.5 Hz, 1H), 7.51 (d, *J* = 9.0 Hz, 1H), 7.02 (d, *J* = 7.2 Hz, 1H), 6.82 (dd, *J* = 9.0, 2.4 Hz, 1H), 6.57 (d, *J* = 2.4 Hz, 1H), 4.35 (s, 3H), 3.75 (t, *J* = 4.7 Hz, 4H), 3.54 (q, *J* = 7.1 Hz, 6H), 2.62 (t, *J* = 6.8 Hz, 2H), 2.56 (t, *J* = 4.7 Hz, 4H), 2.01 (p, *J* = 6.8 Hz, 2H), 1.25 (t, *J* = 7.1 Hz, 6H). ^{19}F NMR (375 MHz, CD_3OD) δ -129.8. ^{13}C NMR (125 MHz, DMSO-d_6) δ 159.3, 156.0, 153.0 (d, *J* = 1.8 Hz), 151.9, 151.0 (d, *J* = 252.7 Hz), 144.8, 143.9 (d, *J* = 14.6 Hz), 141.2 (d, *J* = 5.0 Hz), 139.6, 138.2, 130.3, 120.1 (d, *J* = 10.0 Hz), 118.2, 115.1, 113.2, 111.8 (d, *J* = 20.0 Hz), 109.8, 108.1, 96.0, 94.4 (d, *J* = 4.6 Hz), 65.8 (2C), 56.0, 53.0 (2C), 44.0 (2C), 41.4, 39.1, 23.8, 12.0 (2C). HRMS (ESI) m/z: calcd for $C_{32}H_{38}FN_4O_3^+$ $[M-I]^+$ 545.2922, found 545.2919. HPLC purity: 95.5%.

Q5 (MitoQUMA), yield 31%. ^{1}H NMR (500 MHz, DMSO-d_6) δ 8.59 (d, *J* = 8.5 Hz, 1H), 8.39 (s, 1H), 8.01 (d, *J* = 15.6 Hz, 1H), 7.94 (s, 1H), 7.92 (d, *J* = 3.5 Hz, 1H), 7.70 (d, *J* = 15.6 Hz, 1H), 7.67 (t, *J* = 6.1 Hz, 1H), 7.55 (d, *J* = 8.9 Hz, 1H), 7.07 (d, *J* = 7.1 Hz, 1H), 6.82 (dd, *J* = 9.0, 2.5 Hz, 1H), 6.60 (d, *J* = 2.5 Hz, 1H), 4.32 (s, 3H), 3.57 – 3.48 (m, 6H), 2.77 – 2.59 (m, 10H), 2.41 (s, 3H), 1.94 (p, *J* = 6.7, 6.1 Hz, 2H), 1.19 (t, *J* = 7.0 Hz, 6H). ^{19}F NMR (375 MHz, DMSO-d_6) δ -127.44. ^{13}C NMR (125 MHz, DMSO-d_6) δ 159.4, 156.1, 153.3 (d, $J_{C,F}$ = 2.4 Hz), 151.1 (d, $J_{C,F}$ = 253.1 Hz), 152.0, 145.0, 144.0 (d, $J_{C,F}$ = 14.5 Hz), 141.4 (d, $J_{C,F}$ = 5.1 Hz), 139.7, 138.3, 130.3, 120.3 (d, $J_{C,F}$ = 9.7 Hz), 118.6, 115.3, 113.4, 112.0 (d, $J_{C,F}$ = 20.1 Hz), 109.9, 108.2, 96.1, 94.6 (d, $J_{C,F}$ = 3.6 Hz), 54.7, 52.9 (2C), 50.7 (2C), 44.0 (2C), 43.7, 41.0, 40.1, 23.9, 12.0 (2C). HPLC purity: 96.3%. HRMS (ESI) m/z: calcd for $C_{33}H_{41}FN_5O_2^+$ $[M-I]^+$ 558.3239, found 558.3242.

TPP-QUMA, yield 79%. ^{1}H NMR (400 MHz, CD_3OD) δ 8.59 (d, *J* = 8.7 Hz, 1H), 8.23 (s, 1H), 8.16 (d, *J* = 16.0 Hz, 1H), 8.12 (d, *J* = 9.2 Hz, 1H), 7.96 - 7.72 (m, 17H), 7.51 (d, *J* = 9.1 Hz, 1H), 7.45 (d, *J* = 7.2 Hz, 1H), 6.81 (dd, *J* = 9.0, 2.4 Hz, 1H), 6.51 (d, *J* = 2.4 Hz, 1H), 4.43 (s, 3H), 3.65 – 3.43 (m, 10H), 2.81 (t, *J* = 4.8 Hz, 4H), 2.64 (t, *J* = 7.0 Hz, 2H), 1.95 – 1.85 (m, 2H), 1.85 – 1.74 (m, 2H), 1.25 (t, *J* = 7.1 Hz, 6H). ^{13}C NMR (125 MHz, DMSO-d_6) δ 159.3, 156.2, 154.4 (d, $J_{C,F}$= 1.7 Hz), 153.5 (d, $J_{C,F}$ = 254.4 Hz), 152.2, 145.6, 145.5 (d, $J_{C,F}$ = 14.8 Hz), 141.4 (d, $J_{C,F}$ = 3.6 Hz), 140.1, 137.9, 134.5 (d, $J_{C,P}$ = 2.8 Hz, 3C), 133.2 (d, $J_{C,P}$ = 10.2 Hz, 6C), 130.6, 129.8 (d, $J_{C,P}$ = 12.4 Hz, 6C), 122.5 (d, $J_{C,F}$ = 11.7 Hz), 118.2 (d, $J_{C,P}$ = 85.5 Hz, 3C), 117.8, 117.7, 114.2 (d, $J_{C,F}$ = 23.2 Hz), 113.1, 110.0, 108.2, 104.9 (d, $J_{C,F}$ = 6.0 Hz), 96.1, 55.3, 51.6 (2C), 49.1 (2C), 44.0 (2C), 40.1, 28.4 (d, $J_{C,P}$ = 17.6

Hz), 20.2 (d, $J_{C,P}$ = 49.9 Hz), 19.3 (d, $J_{C,P}$ = 3.2 Hz), 12.0 (2C). ^{19}F NMR (375 MHz, CD_3OD) δ -117.97. ^{31}P NMR (200 MHz, DMSO-d_6) δ 24.10.

1.2 Circular Dichroism Spectroscopy

Circular dichroism (CD) spectra were recorded on a Chirascan circular dichroism spectrophotometer (Applied Photophysics). A quartz cuvette with a 10 mm path length was used to record spectra over a wavelength range of 230 - 330 nm with a 1 nm bandwidth, 1 nm step size, and time of 0.5 s per point. The CD-based real-time assay was performed at a fixed concentration of mtRNA or mtDNA G4s (1 μM) in 10 mM Tris-HCl buffer (100 mM KCl, pH 7.4). The CD melting assay was performed at a fixed concentration of mtRNA or mtDNA G4s (1 μM) in 10 mM Tris-HCl buffer (40 mM KCl, pH 7.4) with or without 1 μM **MitoQUMA**. The data were recorded at intervals of 2.5 °C over a range of 25 - 95 °C, with a heating rate of 1.0 °C/min. The final analysis of the data was conducted using Prism 9.0 (GraphPad Software Inc.).

1.3 Cytotoxicity and Cell Number Analysis

For cytotoxicity assay, cells were seeded in a 96-well plate (5×10^3 cells/well), grew overnight, and treated with various concentrations of **MitoQUMA** for 24h. For cell number analysis, the cells were seeded in a 96-well plate (3×10^3 cells/well) and allowed to adhere overnight. They were then treated with 10 μM ICG-001, 20 μM **MitoQUMA** treatment or transfected with siGRSF1 for 24 h. In both assays, after treatment cells were fixed with 70% ethanol overnight and stained with 0.01 mg/mL propidium iodide (PI) (Selleck, S6874) at room temperature for over 4 h. Then the ImageXpress Micro Confocal microscope (Molecular Devices) high-content imaging platform automatically focused on the fluorescence channel of PI and determined the cell density by counting the nucleus in each well. The IC_{50} was calculated by nonlinear regression fit by GraphPad software with three replicates.

1.4 Plasmid Construction and siRNA

cDNA encoding GRSF1 was obtained by gene synthesis form Addgene. The CDS of GRSF1 was cloned into eGFP-pcDNA 3.1 to generate GFP-tagged protein and eGFP-pcDNA 3.1 was used as the vector control for analysis. Human FASTKD2 gene ORF cDNA clone expression plasmid with N-GFPSpark tag was purchased from Sino Biology (HG16531-ANG). For GRSF1 (5'-

CGGUUCUUAUAAGGGAAAGAATT-3') knockdown and FASTKD2 (5'-CAATGAGTGTGATGAGATA-3'), the synthesized duplex RNAi oligos targeting human mRNA sequence from RIBOBIO were used. A scrambled duplex RNA oligo (5'-UUCUCCGAACGUGUCACGUTT-3') was used as RNA control. Twenty-four hours before transfection, the medium was replaced with fresh medium and transfected using Lipofectamine 3000 reagent (Invitrogen) with vector control, plasmid construct, siRNA negative control (siNC). The working concentration of siRNA was 50 nM.

1.5 Immunofluorescence

Cells were fixed in 4% paraformaldehyde/PBS at room temperature for 15 min, then washed in PBS and permeabilized in 0.5% Trition X-100/PBS at 37 °C for 15 min. Then cells were blocked in 5% BSA/PBS at 37 °C for 1 h. Subsequently, cells were incubated with primary antibody overnight at 4 °C. After PBS washes, secondary antibodies were applied and incubated for 45 min at room temperature. DAPI was used to indicate nucleus. All immunoblotting was performed using the indicated primary antibodies, including anti-GRSF1 (abcam, ab205531, 1:1000 dilution), anti-FASTKD2 (proteintech, 17464-1-AP, 1:1000 dilution), anti-Actin (proteintech, 66009-1-lg, 1:1000 dilution) , anti-Histone H3 (abcam, ab176843, 1:1000 dilution), Alexa 488-conjugated antibody (Thermo Fisher Scientific, A0208, 1:3000 dilution) and Alexa 647-conjugated antibody (Thermo Fisher Scientific, A32733, 1:3000 dilution). Digital images were recorded using an FV3000 laser scanning confocal microscopy (Olympus) with a 60 × objective lens. The images were analyzed with Imaris software (Bitplane Corp.).

1.6 RNA Extraction and Real-time PCR

Cells were collected and the total RNA was extracted by the HiPure Total RNA Mini kit (Magen, R4111) with gDNA filter and then reverse-transcribed into complementary DNA (cDNA) by Evo M-MLV RT kit (Accurate Biotechnology, AG11705). The mRNA level of exact genes was measured by qPCR of cDNA by assessing the relative levels of the exact gene versus the reference gene (Actin). The qPCR analysis was performed using SYBR Green Premix Pro Taq HS qPCR Kit (Accurate Biotechnology, AG11701) with the LightCycler480 (Roche) system. Primer sequences for quantitative RT-PCR are provided in Table S2.

1.7 Western Blot Analysis

Cells were lysed with RIPA buffer containing 1 mM PMSF and 1 mM DTT. The concentration of protein was calculated with a BCA protein assay kit. Then, the protein was denatured with 1× protein loading dye heating at 95 °C for 10 min. An equal amount of protein samples was electrophoresed on 10% SDS-PAGE gel at 100 V for 2 h and transferred to a nitrocellulose membrane at 300 mA for 1.5 h. The membranes were blocked with 5% BSA for 1 h at room temperature and incubated with primary antibodies overnight at 4 °C, followed by secondary antibodies for 1 h at room temperature. Signal detection was performed using a chemiluminescence imaging system (Tanon-4600). All immunoblotting was performed using the indicated primary antibodies, include anti-GRSF1 (abcam, ab205531, 1:1000 dilution), anti-GAPDH (proteintech, 60004-1, 1:1000 dilution), anti-β-catenin (abcam, ab6302, 1:1000 dilution) and HRP-conjugated secondary antibodies (beyotime, A0208 and A0216, 1:3000 dilution).

2. Supplementary Materials

Table S1. Sequence of oligomers used in the study

Name	Sequence (5' to 3')	Structure in K+ solution
Mito3	r(CGGGGGGAGGGGGGGUUUGGUGGA)	G-quadruplex
Mito125	r(UGGGCUAUUUUCUGCUAGGGGGUGGAAG)	G-quadruplex
Mito126	r(GGGAGGUUGAAGUGAGAGGUAUGGU)	G-quadruplex
Mito152	r(GAAUUUUGGGGGAGGUUAUAUGGG)	G-quadruplex
Mito155	r(GGGGGUUAAUUUUGCGUAUUGGGG)	G-quadruplex
SS12	r(AUACGAUGCUUA)	Single-strand
HP18	r(CAGUACAGAUCUGUACUG)	Duplex
Mito27	d(AGGTCGGGGCGGTGATGTAGAGGGTGAT GGT)	G-quadruplex
Mito130	d(TGGCGTTTAATGGGGTTTAGTAGGGTGGGG)	G-quadruplex
Mito0.5-22	d(GGTTAGGCGTACGGCCAGGGCTATTGG)	G-quadruplex

Table S2. PCR primers used in the present study

Primer name	Sequence (5' to 3')	Gene	Target
Actin-F	CACCATTGGCAATGAGCGGTTC	Actin	mRNA
Actin-R	AGGTCTTTGCGGATGTCCACGT	Actin	mRNA
GRSF1-F	TGGAGTCAGAGCAGGATGTGCA	GRSF1	mRNA
GRSF1-R	GGCGAAGATTTGACCTGCAAGC	GRSF1	mRNA
ND1-F	TAATGCTTACCGAACGAA	ND1	mRNA
ND1-R	GGTGATGGTAGATGTGGC	ND1	mRNA
ND2-F	GCCCAACCCGTCATCTAC	ND2	mRNA
ND2-R	TTGCTTGCGTGAGGAAAT	ND2	mRNA
COX1-F	CCCCGCATAAACAACATA	COX11	mRNA
COX1-R	AGGACGGATCAGACGAAG	COX1	mRNA
COX2-F	ACCTACGAGTACACCGACTA	COX2	mRNA
COX2-R	TTGCTCCACAGATTTCAG	COX2	mRNA
ATP8-F	ACCTACCTCCCTCACCAA	ATP8	mRNA
ATP8-R	GCAATGAATGAAGCGAAC	ATP8	mRNA
ATP6-F	AGCGGGCACAGTGATTAT	ATP6	mRNA
ATP6-R	AGGCGACAGCGATTTCTA	ATP6	mRNA
COX3-F	GAAGTCCCACTCCTAAACA	COX3	mRNA
COX3-R	TATTAGTTGGCGGATGAA	COX3	mRNA
ND3-F	CAAACAACTAACCTGCCACT	ND3	mRNA
ND3-R	TTCGGTTCAGTCTAATCCTT	ND3	mRNA
ND4L-F	TCGCTCACACCTCATATCCTC	ND4L	mRNA
ND4L-R	AGGCGGCAAAGACTAGTATGG	ND4L	mRNA
ND4-F	TTCCCCAACCTTTTCCTCCG	ND4	mRNA
ND4-R	TGGATAAGTGGCGTTGGCTT	ND4	mRNA
ND5-F	GCCCTTCTAAACGCTAAT	ND5	mRNA
ND5-R	GCTGCGAACAGAGTGGTG	ND5	mRNA
ND6-F	TTCTTCTAAGCCTTCTCC	ND6	mRNA
ND6-R	CATACTCTTTCACCCACAG	ND6	mRNA
CYTB-F	CCTGAAACATCGGCATTA	CYTB	mRNA
CYTB-R	GGGTGGGACTGTCTACTG	CYTB	mRNA
lncND5-F	GGCAGGTTTTGGCTCGTAAGAAG	lncND5	lncRNA
lncND5-R	CAACTACCTAACCAACAAACTTA	lncND5	lncRNA
lncCYTB-F	GGTCTGCGGCTAGGAGTCAATA	lncCYTB	lncRNA
lncCYTB-R	TTTCGCCCACTAAGCCAATCA	lncCYTB	lncRNA

3. Other Supporting Spectra and Graphs

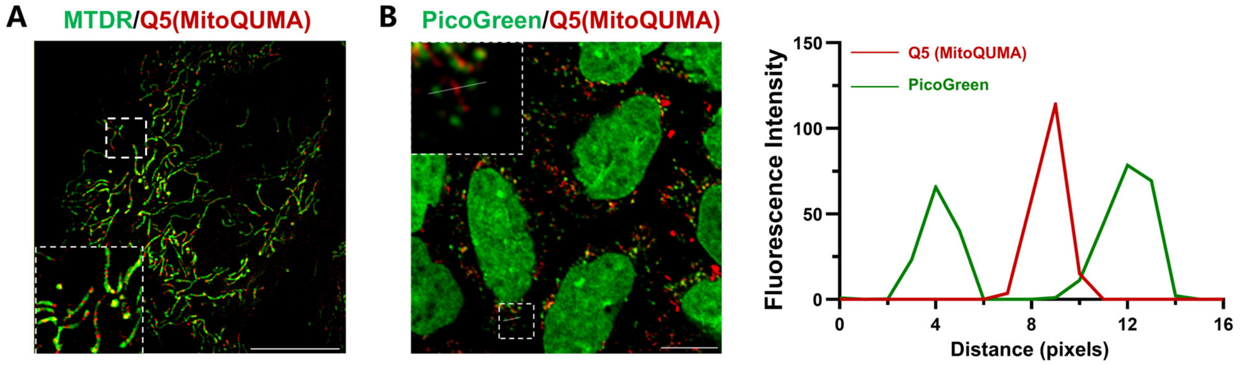

Figure S1. Super-resolution and confocal imaging confirm mitochondrial localization of MitoQUMA in live HeLa cells. (A) Super-resolution image of live HeLa cells co-stained with 50 nM MitoTracker Deep Red (MTDR, λex = 640 nm) and 2 μM **Q5 (MitoQUMA)** (λex = 561 nm). (B) Confocal image of live HeLa cells co-stained with 0.1 μM PicoGreen and 2 μM **Q5 (MitoQUMA)**. Fluorescence intensity profiles across the white line in the white box are shown. Scale bars for the cell image: 10 μm.

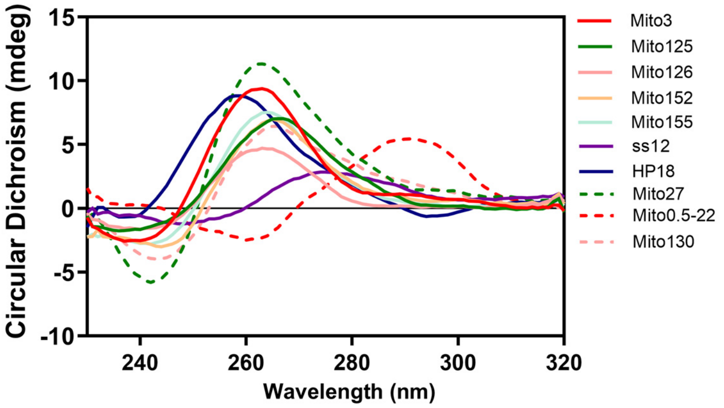

Figure S2. The CD spectra of 1 μM mtRNA and mtDNA G4s in 10 mM Tris-HCl buffer, 100 mM KCl, pH 7.4.

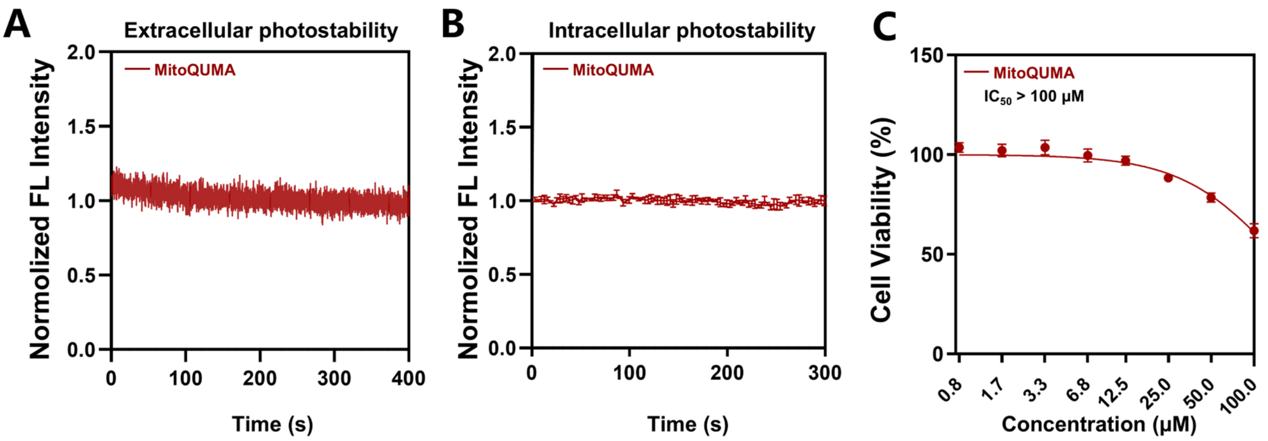

Figure S3. Photostability and cytotoxicity of MitoQUMA. (A) The fluorescence intensity of 2 μM **MitoQUMA** (λem = 630 nm) in PBS buffer (pH=7.4) under continuous excitation at 555 nm. (B) Quantitative analysis of changes in fluorescence intensity of 2 μM **MitoQUMA** in live HeLa cells under continuous excitation at 561 nm. (C) The impact of **MitoQUMA** (0-100 μM) on the viability of HeLa cells after 24 h of treatment. Biological replicates (n = 3) were taken. The data are presented as mean ± SEM.

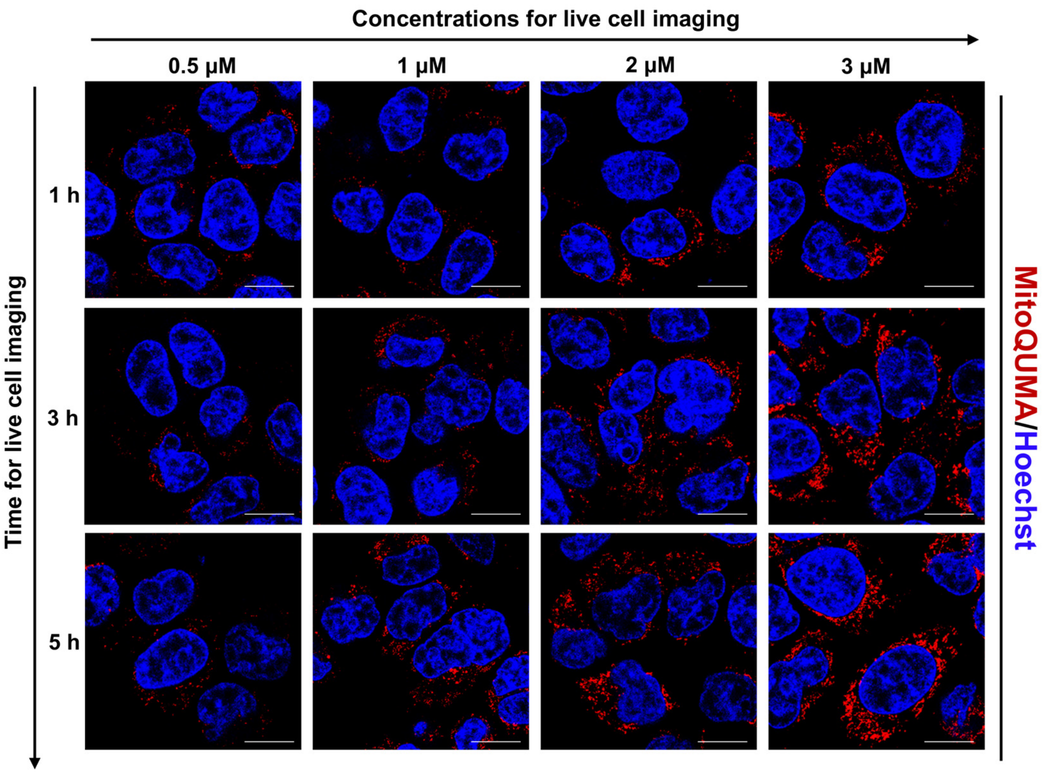

Figure S4. Live HeLa cells were stained with 0.5-3 μM **MitoQUMA** (λex = 561 nm, red) for 1-5 h. The nucleus is indicated by Hoechst33342 (blue). Scale bars for cell image: 10 μm.

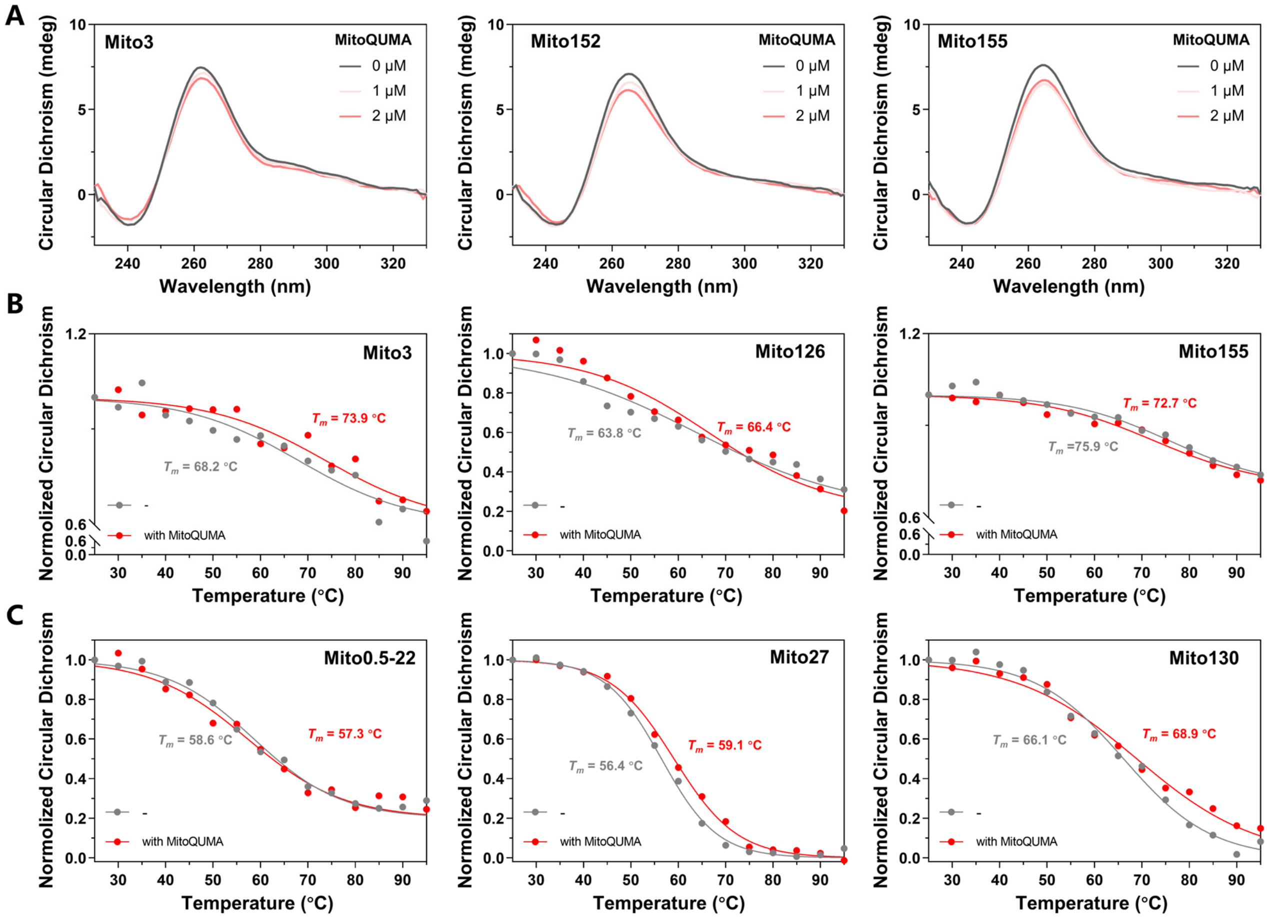

Figure S5. (A) The CD spectra of 1 μM mtRNA G4 (Mito3, Mito152, Mito155) with the stepwise addition of **MitoQUMA** (0-2 μM) in buffer containing low K^+ concentration (10 mM Tris-HCl, 2 mM KCl, pH 7.4). (B) The CD melting curves of 1 μM mtRNA G4 (Mito3, Mito126, Mito155) with or without 1 μM **MitoQUMA** in 10 mM Tris-HCl buffer, 40 mM KCl, pH 7.4. (C) The CD melting curves of 1 μM mtDNA G4 (Mito0.5-22, Mito27, Mito130) with or without 1 μM **MitoQUMA** in 10 mM Tris-HCl buffer, 40 mM KCl, pH 7.4.

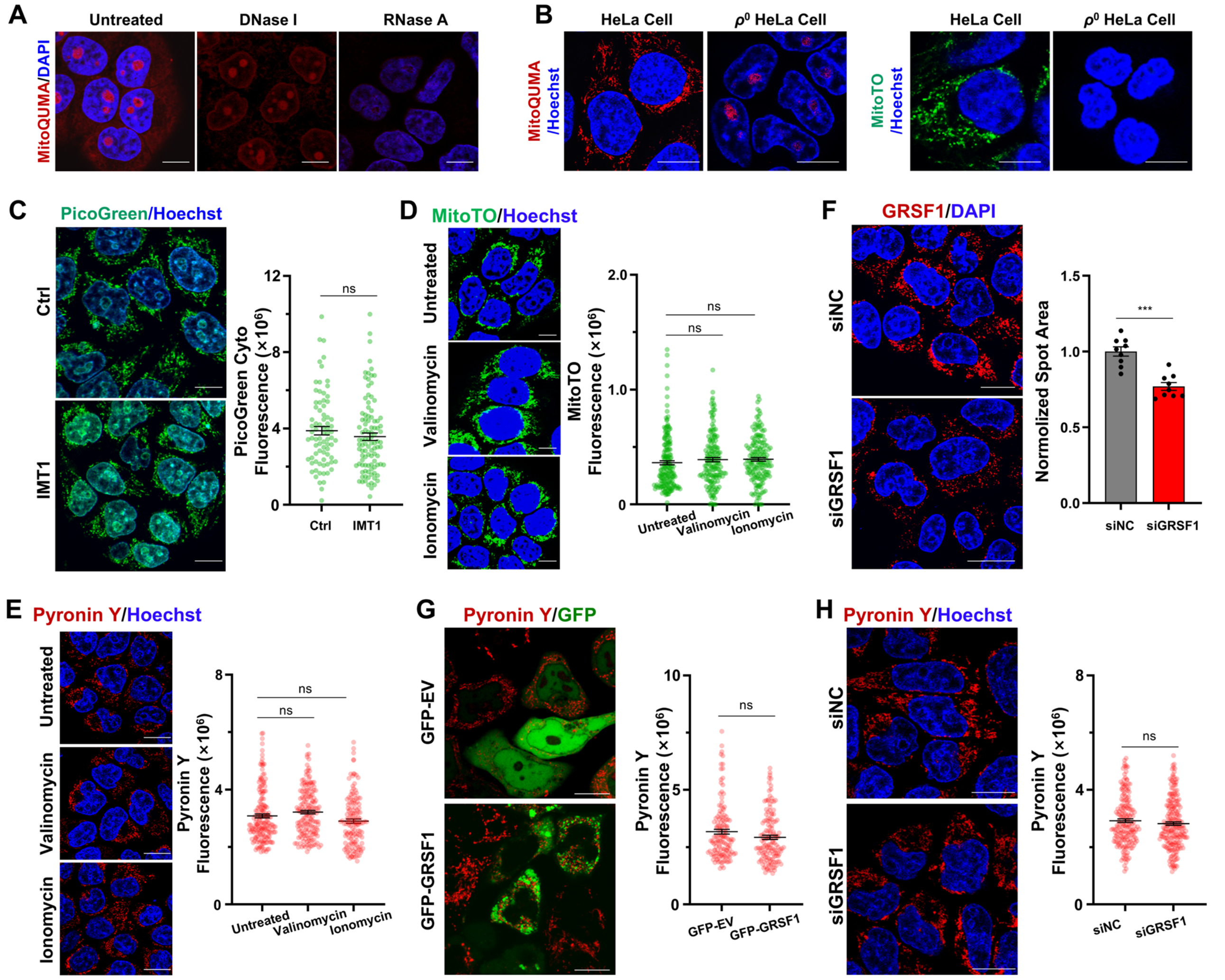

Figure S6. **Validation of RNA selectivity, mitochondrial RNA targeting, and functional relevance of MitoQUMA**. (A) HeLa cells stained with **MitoQUMA**. The fluorescence remains after DNase I treatment and complete loss of **MitoQUMA** fluorescence after RNase A treatment. (B) Live HeLa cells and ρ^0 HeLa cells were stained with 2 μM **MitoQUMA** (red) and MitoTO (green). The nucleus was visualized by Hoechst33342 (blue). (C) Live HeLa cells were treated with 5 μM IMT1 followed by stained with 0.1 μM PicoGreen. (D) Live HeLa cells were treated with valinomycin (together with 200 mM KCl) or ionomycin (together with 20 mM KCl), and stained with 2 μM MitoTO. (E) Live HeLa cells were treated with valinomycin (together with 200 mM KCl) or ionomycin (together with 20 mM KCl), and stained with 1 μM Pyronin Y. (F) HeLa cells were transfected with siRNA to knock down GRSF1 expression, followed by immunofluorescence staining of GRSF1. (G) Live HeLa cells with overexpression of GFP-tagged GRSF1 were stained with 1 μM Pyronin Y. (H) Live HeLa cells were transfected with siRNA to knock down GRSF1 expression, followed by staining with 1 μM Pyronin Y. For each sample of cell image, approximately 100 cells were measured. Biological replicates (n = 3) were taken. The data are presented as mean ± SEM, and statistical significance is determined by the two-sided Student's unpaired t-test (C, E, G and H) and one-way ANOVA followed by Dunnett's multiple comparisons test (D and F) as (ns) not significant, (*) $P < 0.05$, (**) $P < 0.01$, and (***) $P < 0.001$. Scale bars for cell image: 10 μm.

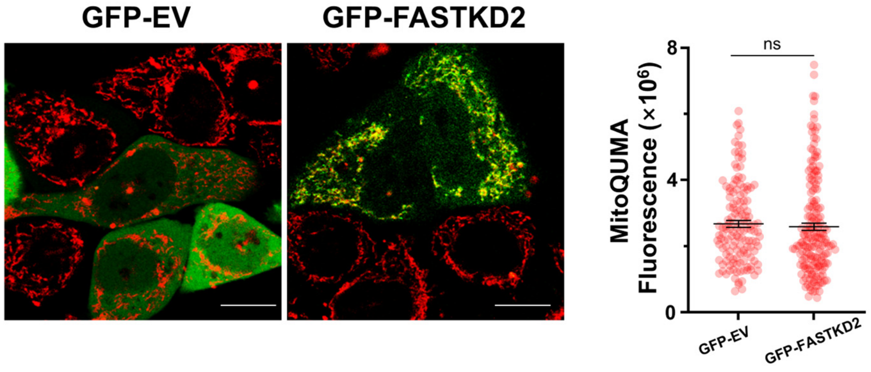

Figure S7. Live HeLa cells expressing GFP-tagged vector (GFP-EV) and FASTKD2 (GFP-FASTKD2) underwent staining with 2 μM **MitoQUMA**. For each sample of cell image, approximately 100 cells were measured. Biological replicates (n = 3) were taken. The data are presented as mean ± SEM, and statistical significance is determined by the two-sided Student's unpaired t-test as (ns) not significant, (*) $P < 0.05$, (**) $P < 0.01$, and (***) $P < 0.001$. Scale bars for cell image: 10 μm.

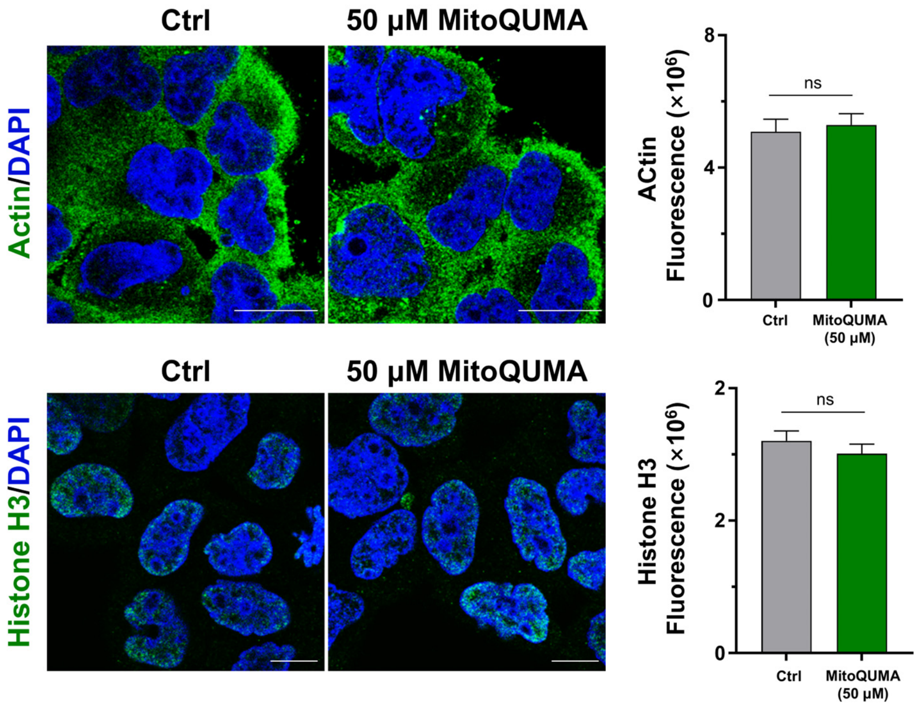

Figure S8. HeLa cells were treated with 50 μM **MitoQUMA** for 24 h, followed by immunofluorescence staining of Actin and Histone H3. For each sample of cell image, approximately 100 cells were measured. Biological replicates (n = 3) were taken. The data are presented as mean ± SEM, and statistical significance is determined by the two-sided Student's unpaired t-test as (ns) not significant, (*) $P < 0.05$, (**) $P < 0.01$, and (***) $P < 0.001$. Scale bars for cell image: 10 μm.

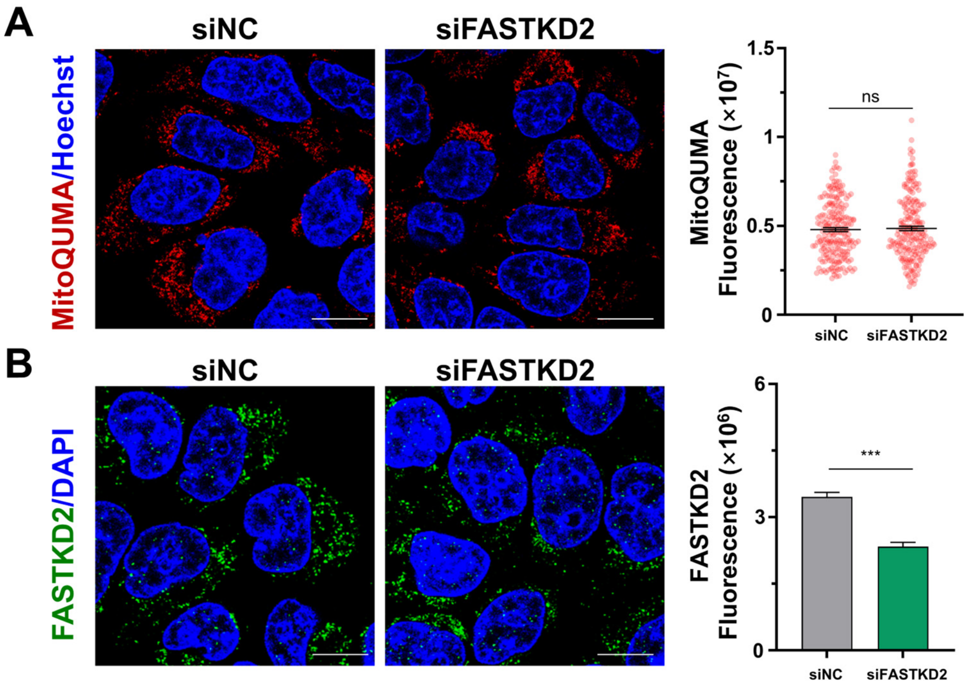

Figure S9. (A) Live HeLa cells were transfected with siRNA to knock down FASTKD2 expression, followed by staining with 2 μM **MitoQUMA**. (B) HeLa cells were transfected with siRNA to knock down FASTKD2 expression, followed by immunofluorescence staining of FASTKD2. For each sample of cell image, approximately 100 cells were measured. Biological replicates (n = 3) were taken. The data are presented as mean ± SEM, and statistical significance is determined by the two-sided Student's unpaired t-test as (ns) not significant, (*) $P < 0.05$, (**) $P < 0.01$, and (***) $P < 0.001$. Scale bars for cell image: 10 μm.

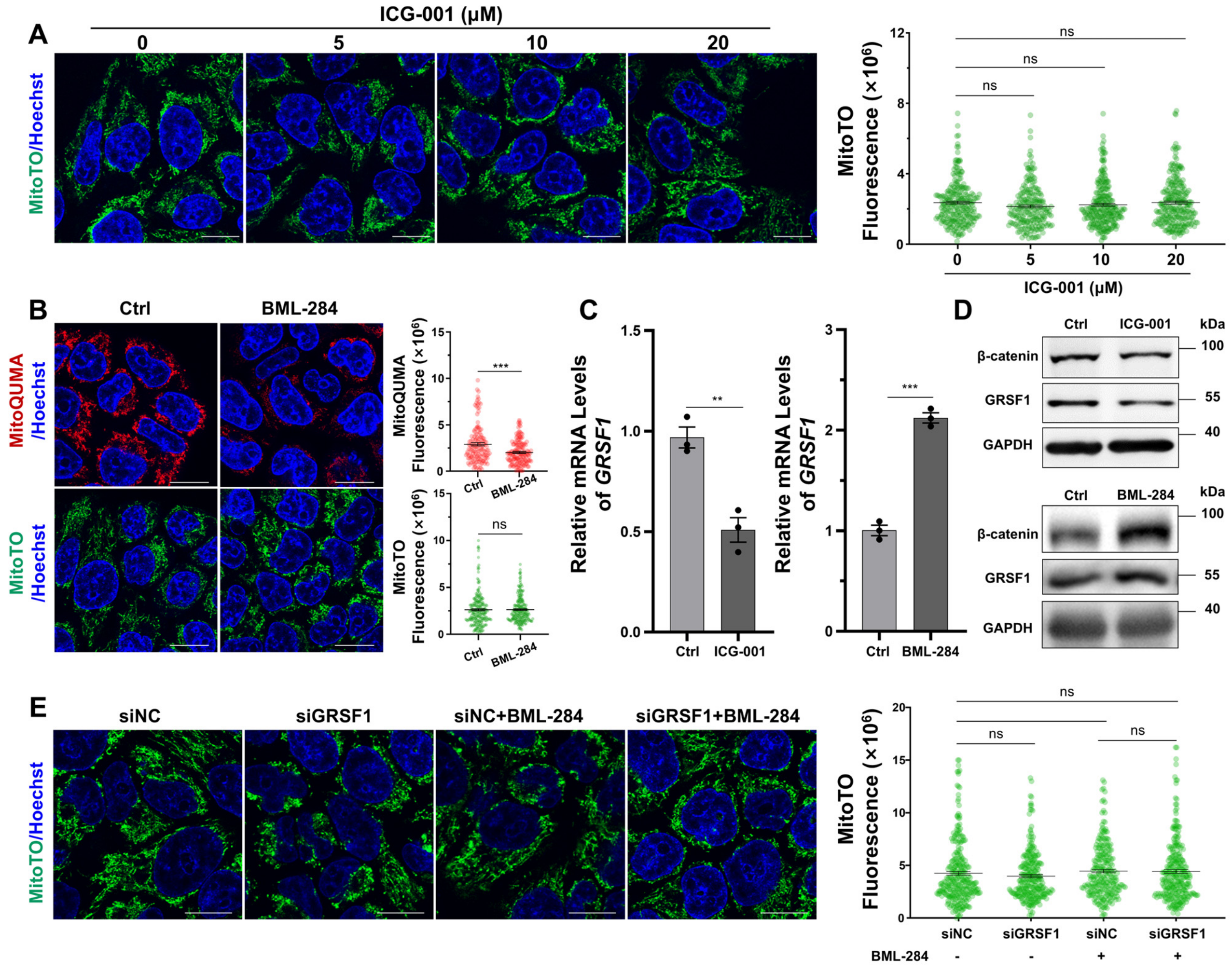

Figure S10. **The Wnt/β-catenin-GRSF1 signaling axis regulates mitochondrial RNA granule dynamics.** (A) Live HeLa cells were treated with varying concentrations of ICG-001 treatment, followed by staining with 2 μM MitoTO. (B) Live HeLa cells were treated with 20 nM BML-284, followed by stained with 2 μM MitoTO. (C) The impact of 10 μM ICG-001 and 20 nM BML-284 treatment on GRSF1 mRNA expression. (D) The impact of 10 μM ICG-001 and 20 nM BML-284 treatment on GRSF1 protein expression. (E) Live HeLa cells were transfected with siRNA to knock down GRSF1 expression and then subjected with or without 20 nM BML-284, followed by staining with 2 μM MitoTO. For each sample of cell image, approximately 100 cells were measured. Biological replicates (*n* = 3) were taken. The data are presented as mean ± SEM, and statistical significance is determined by the two-sided Student's unpaired *t*-test (B and C) and one-way ANOVA followed by Dunnett's multiple comparisons test (A and E) as (ns) not significant, (*) $P < 0.05$, (**) $P < 0.01$, and (***) $P < 0.001$. Scale bars for cell image: 10 μm.

NMR, HRMS and HPLC spectra of the final compounds

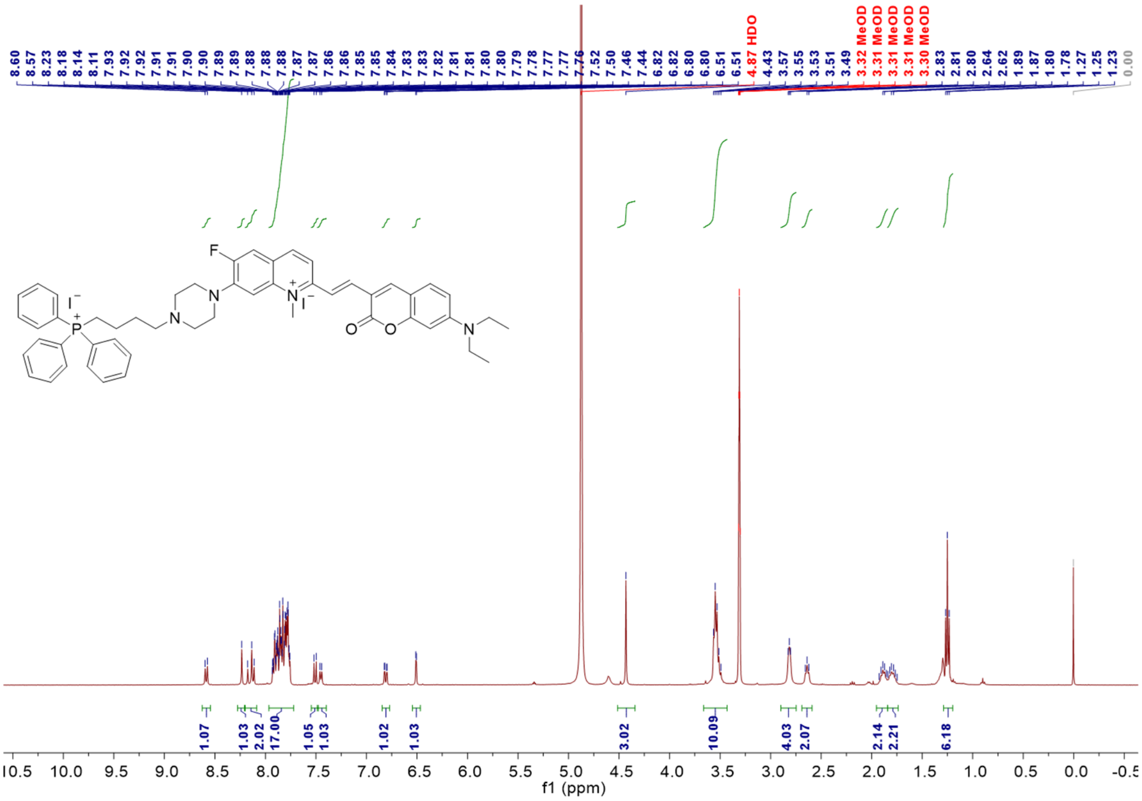

^{1}H NMR spectrum of **TPP-QUMA.**

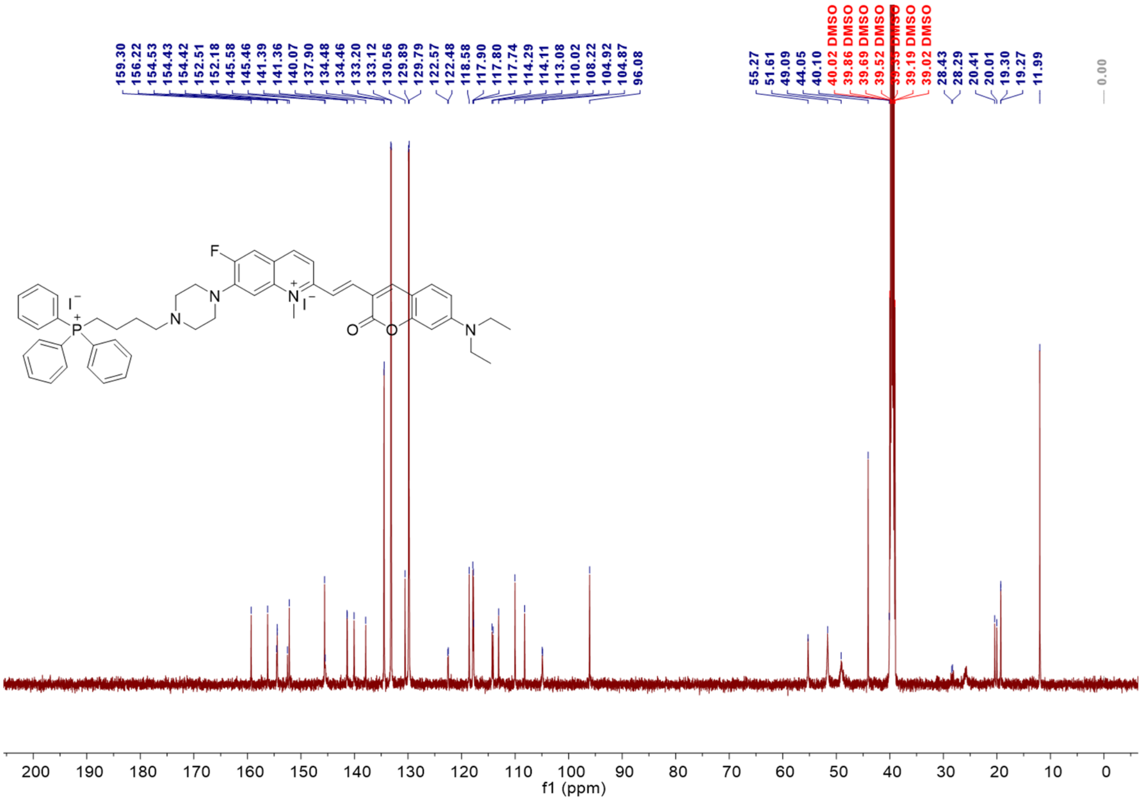

^{13}C NMR spectrum of **TPP-QUMA.**

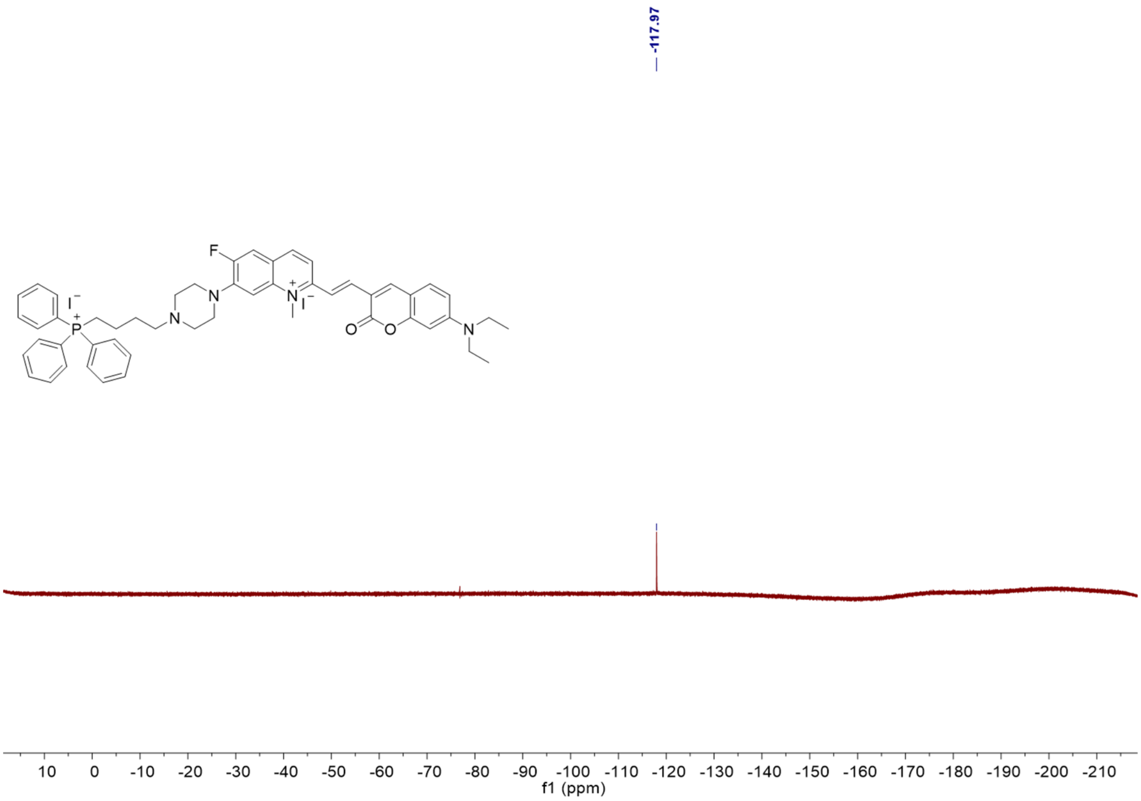

^{19}F NMR spectrum of **TPP-QUMA**.

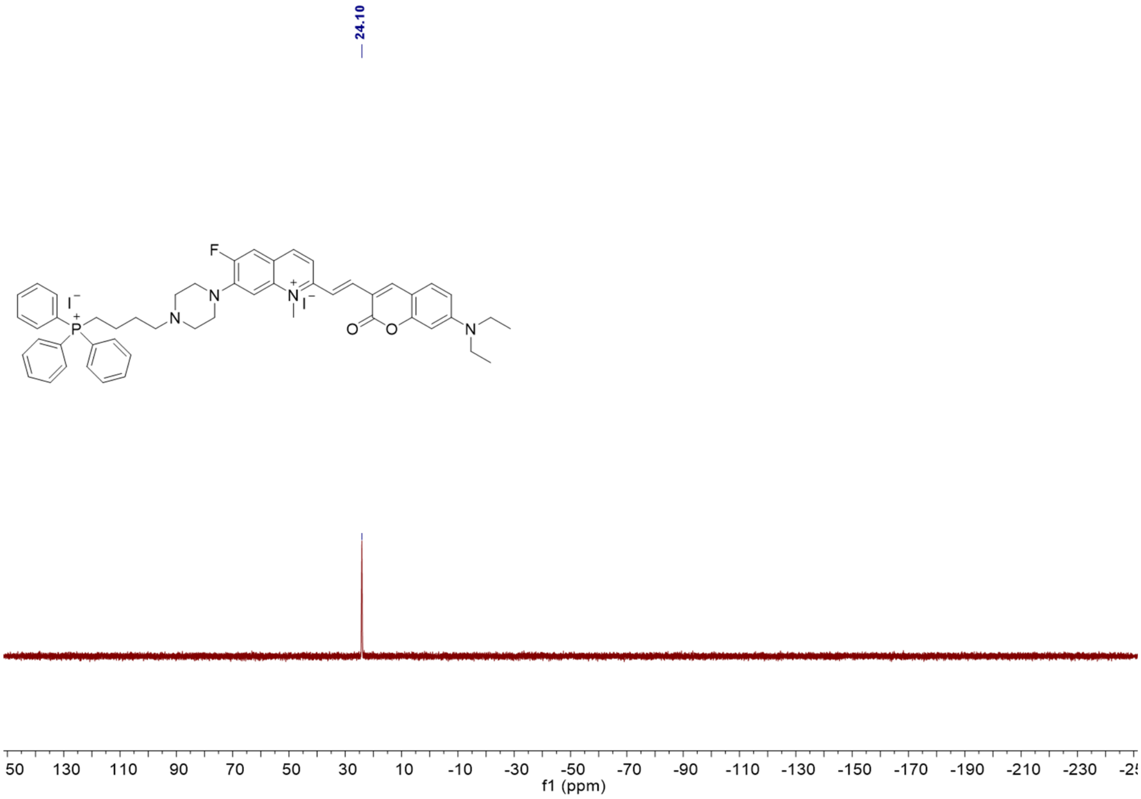

^{31}P NMR spectrum of **TPP-QUMA**.

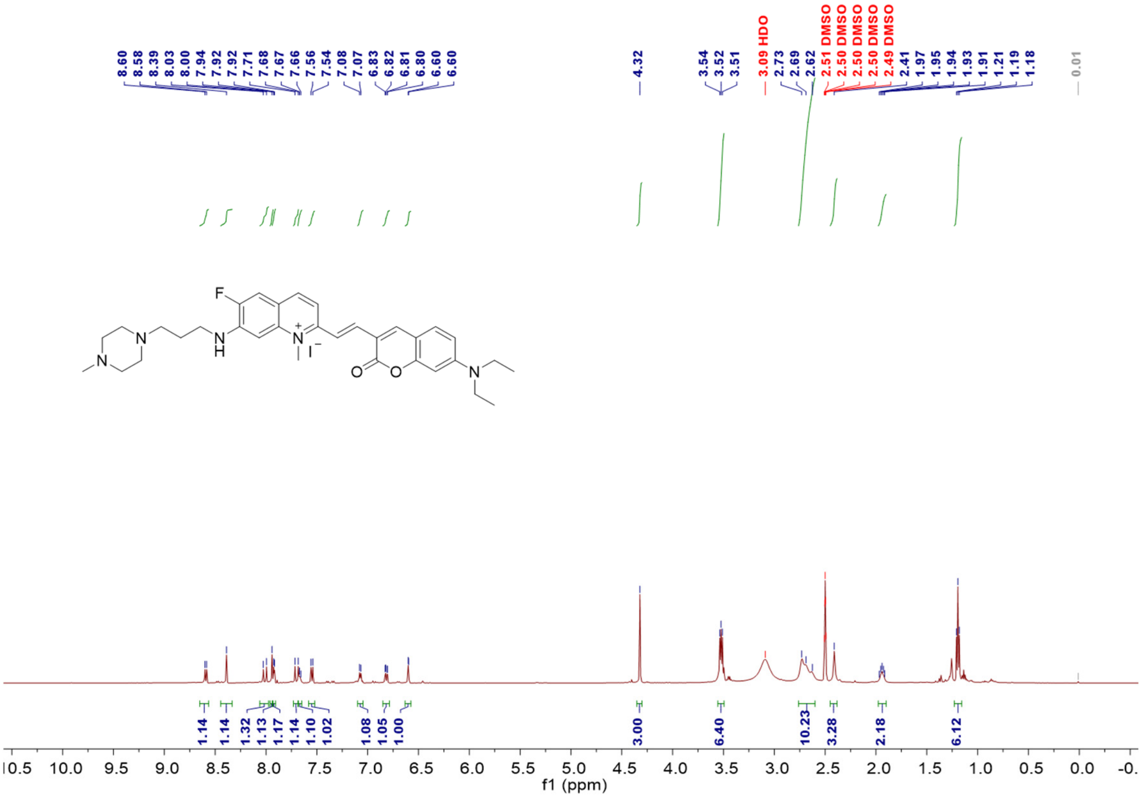

^{1}H NMR spectrum of **MitoQUMA**.

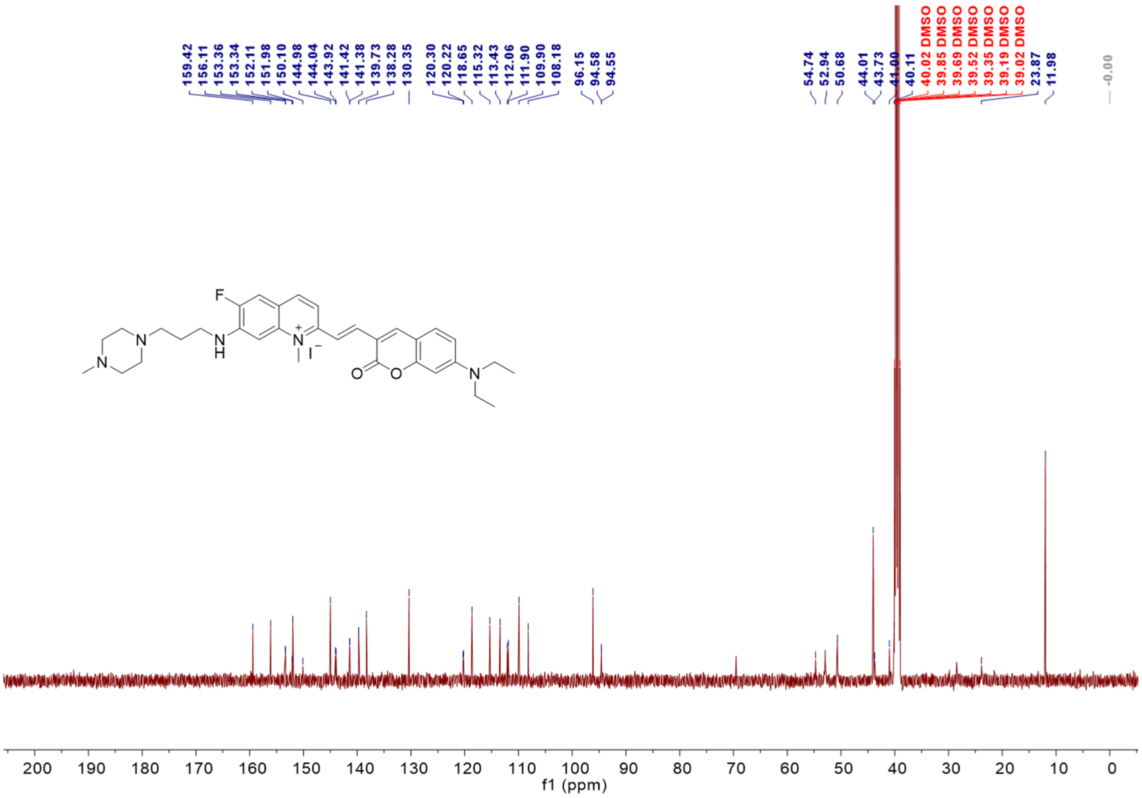

^{13}C NMR spectrum of **MitoQUMA**.

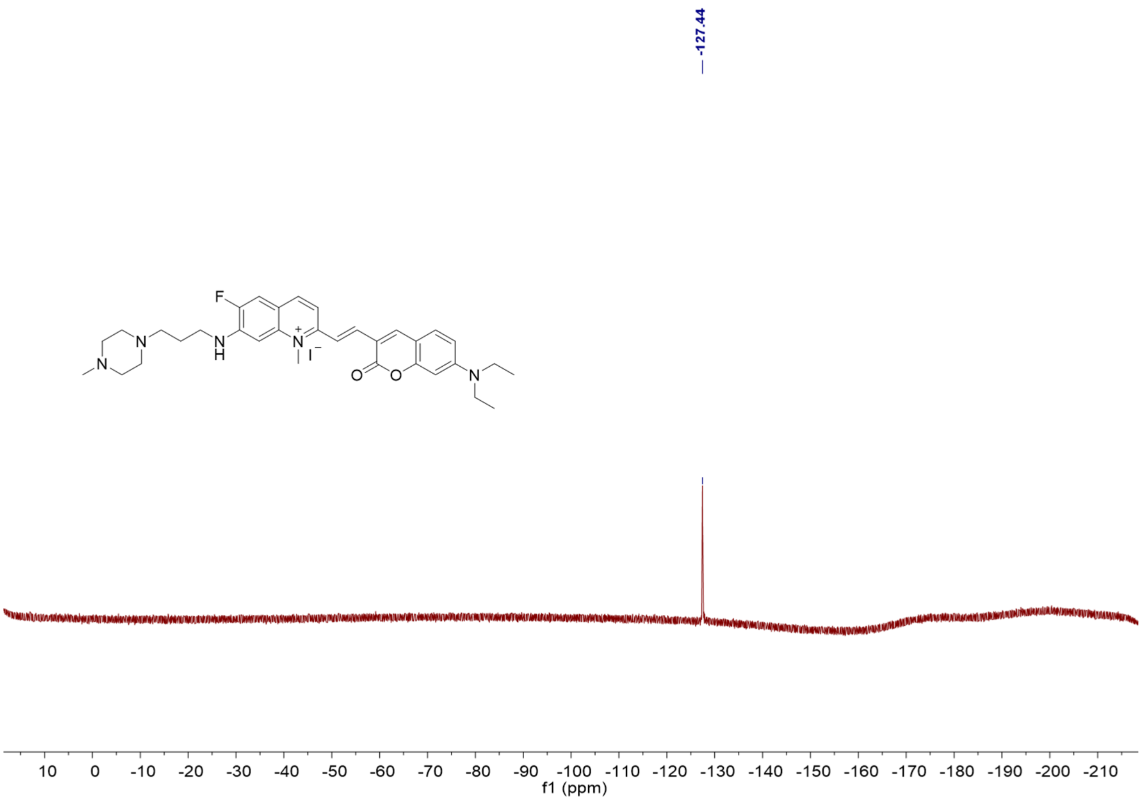

^{19}F NMR spectrum of **MitoQUMA**.

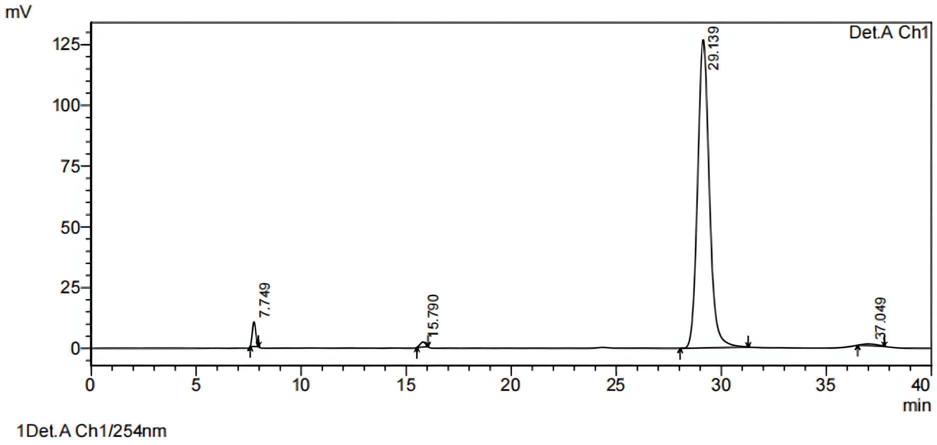

Detector A Ch1 254nm

Peak#	Ret. Time	Area	Height	Area %	Height %
1	7.749	117289	10088	2.304	7.223
2	15.790	35764	2049	0.702	1.467
3	29.139	4905415	126831	96.344	90.805
4	37.049	33095	706	0.650	0.506
Total		5091563	139674	100.000	100.000

The HPLC analysis of **MitoQUMA**.

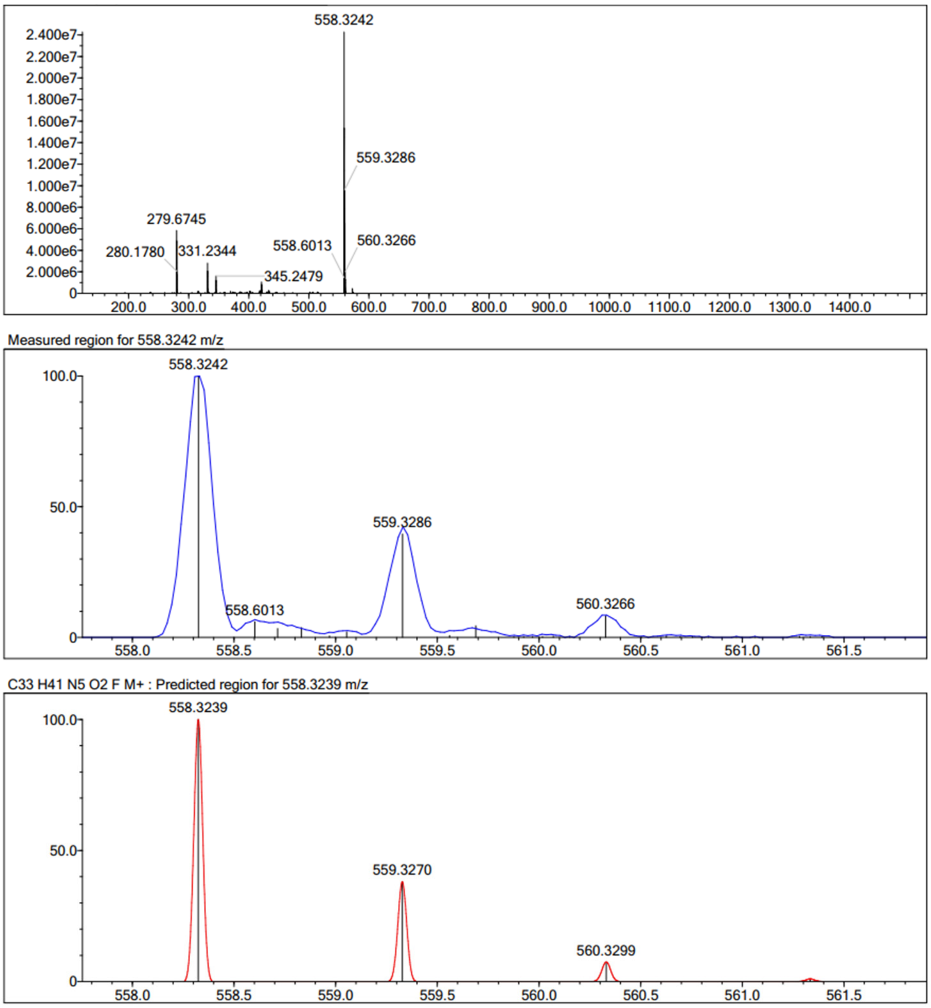

Rank	Score	Formula (M)	Ion	Meas. m/z	Pred. m/z	Df. (mDa)	Df. (ppm)	Iso	DBE
1	84.59	C33 H41 N5 O2 F	M+	558.3242	558.3239	0.3	0.54	84.59	15.5

The HRMS spectrum of **MitoQUMA**.

4. Reference

1. Li, M. L.; Dai, L. T.; Gao, Z. Y.; Yan, J. T.; Xu, S. M.; Tan, J. H.; Huang, Z. S.; Chen, S. B.; Chen, X. C., Discovery of Novel Coumarin-quinolinium Derivatives as Pan-KRAS Translation Inhibitors by Targeting 5'-UTR RNA G-Quadruplexes. *J. Med. Chem.* **2024,** *67* (3), 1961-1981.
2. Chen, X. C.; Chen, S. B.; Dai, J.; Yuan, J. H.; Ou, T. M.; Huang, Z. S.; Tan, J. H., Tracking the Dynamic Folding and Unfolding of RNA G-Quadruplexes in Live Cells. *Angew. Chem. Int. Ed.* **2018,** *57* (17), 4702-4706.
